# Exercise training is associated with changes in adipocyte size and lipid metabolism in older women: cross-sectional and longitudinal study

**DOI:** 10.1186/s12877-026-07752-9

**Published:** 2026-06-23

**Authors:** Marek Wilhelm, Terezie Čížková, Klára Daďová, Michal Koc, Jana Neubert, Ondřej Kuda, Jan Gojda, Lenka Rossmeislová, Michaela Šiklová

**Affiliations:** 1 Department of Pathophysiology, Centre for Research on Diabetes, Metabolism and Nutrition, Third Faculty of Medicine, Charles University, Prague, Czech Republic; 2 Faculty of Physical Education and Sport, Charles University, Prague, Czech Republic; 3 Institute of Physiology of the Czech Academy of Sciences, Prague, Czech Republic; 4 Department of Internal Medicine, Third Faculty of Medicine, Charles University, Kralovske Vinohrady University Hospital, Prague, Czech Republic; 5 Franco-Czech Laboratory for Clinical Research on Obesity, Third Faculty of Medicine, Charles University, Prague and Universite Toulouse III – Paul Sabatier (UPS), Toulouse, France

**Keywords:** Exercise training, Adipose tissue, Older women, Adipogenesis, Lipid metabolism

## Abstract

Adaptations in subcutaneous adipose tissue (SAT) metabolism may contribute to cardiometabolic health improvements in response to exercise training, especially during aging. Here we investigated the potential effects of long- and short-term exercise on adipogenesis and lipid metabolism in adipose tissue of older women (age 61–80 years). The effects of long-term (> 5 years) physical activity and short-term (4 months) exercise training were evaluated, with a focus on adipocyte size, adipogenic capacity, gene expression related to lipolysis, lipogenesis, and peroxisomal β-oxidation, along with lipidomic and proteomic analysis.

The difference between long-term physically active/physically fit (*Trained*) and less physically active (*Sedentary*) older women was analyzed in cross-sectional study (*Sedentary*, *n* = 29: age 72 ± 4 yrs, BMI 25.9 ± 2.6 kg/m^2^; *Trainedn* = 28: age 69 ± 4 yrs, BMI 25.1 ± 2.3 kg/m^2^). The effect of short-term exercise training (ET) was observed during 4-months of combined ET in group of 23 women (age 70 ± 4 yrs, BMI 27.2 ± 4.1 kg/m^2^).

Both physical activity durations were associated with reduced adipocyte hypertrophy, which was driven by changes in adiposity. No significant changes in adipose capacity to differentiate was observed. Long-term physical activity increased mRNA expression of β-adrenergic receptors (ADRB1, ADRB2) and elevated very-long chain fatty acid (VLC-FA) levels, correlating positively with cardiorespiratory fitness (VO_2, peak_). Altogether, these findings suggest novel hypotheses regarding SAT metabolic adaptations that may support metabolic health in older women, warranting further investigation in larger cohorts.

**New & noteworthy**

Our study reveals that SAT adipogenic capacity remains largely unaffected by physical activity in older women. However, the observed link between elevated β-adrenergic receptor expression, VLC-FA levels, and fitness suggests potential adipose tissue-specific adaptations that might mediate the positive effects of exercise in older age.

**Supplementary Information:**

The online version contains supplementary material available at https://doi.org/10.1186/s12877-026-07752-9.

## Introduction

Physical activity is widely recognized for its beneficial effects on health, particularly in mitigating age-related declines in metabolic health, cognitive function, and skeletal muscle mass [1]. The metabolic benefits of exercise training (ET) have traditionally been attributed primarily to direct adaptations in skeletal muscle, including enhanced oxidative capacity and improved glucose uptake [2, 3]. However, accumulating evidence over the past decade has highlighted subcutaneous adipose tissue (SAT) as an important and active mediator of exercise-induced metabolic improvements [4, 5]. Both animal and human studies have demonstrated that SAT responds to ET through reduced inflammation, enhanced angiogenesis and vascularization, altered glucose and fatty acid handling, and extracellular matrix remodeling [6–9]. Transcriptomic and proteomic analyses further suggest that physical activity may influence adipogenic capacity and lipid metabolic pathways within adipose tissue [10–14].

High adipogenic capacity is considered a key determinant of adipose tissue expandability, limiting adipocyte hypertrophy and thereby preserving a metabolically healthy adipose tissue phenotype [15, 16]. In parallel, tightly regulated lipid metabolism, including lipolysis, lipid synthesis, and fatty acid oxidation, is essential for proper SAT function and whole-body lipid homeostasis [17, 18]. These regulatory mechanisms are particularly vulnerable to age-related dysregulation [19], suggesting that ET may exert especially important protective effects in older individuals.

Despite a growing body of evidence, the direct impact of physical activity on adipogenic capacity and lipid metabolism in primary human adipocytes remains poorly understood, especially in the aging population. Furthermore, the extent to which adiposity may modulate SAT responsiveness to exercise training (ET) has not been systematically explored, despite its clear clinical relevance.

Accordingly, this study aimed to provide a detailed characterization of SAT adaptations in older women through two complementary approaches: a cross-sectional comparison of long-term physically active and less physically active (sedentary) individuals, and a 4-month longitudinal intervention with ET. Next focus of our analysis was to examine how adiposity, assessed as fat mass (FM), might interact with these SAT characteristics. By integrating clinical data with cellular-level analyses, we sought to identify novel SAT-derived factors associated with changes in insulin sensitivity and cardiorespiratory fitness (VO₂, _peak_), thereby highlighting potential adipose tissue-specific pathways that may support metabolic health during aging.

## Materials and methods

The study is part of the clinical trial registered on December 18, 2017 as EXODYA (Effect of EXercise training and Omega-3 fatty acids on metabolic health and DYsfunction of Adipose tissue in older, No: NCT03386461, clinicaltrials.org). The primary objective of the clinical trial was to study the effects of ET and Calanus oil supplementation on inflammatory parameters of SAT, the outcomes of the present study are explorative.

The recruitment, exclusion criteria and setting of the study were published before [6]. In brief, older women aged 61–80 years were included in the study. Participants for the study were recruited mainly through advertisements in senior citizens’ clubs and universities of the third age. All clinical examinations were performed at the Královské Vinohrady University Hospital. Exclusion criteria were: weight loss of more than 3 kg during the 3 months prior to the study; smoking; diagnosed cancer, diabetes, or liver/kidney disease; untreated hypothyroidism; use of beta-blockers, steroids, and/or omega-3 supplements; and long-term use of nonsteroidal anti-inflammatory drugs (anti-rheumatic drugs and analgesics affecting cyclooxygenase; 100 mg of aspirin per day was considered acceptable). Patients taking cholesterol-lowering and blood pressure-lowering medications (representing 70% to 90% of the elderly population) were included in the study. Participants provided 3-day dietary records before the start of the study and at the end of exercise training (ET) and were instructed not to change their dietary habits during the study.

The study was divided into two parts (Suppl. Material 1): (i) Study I: Long-term ET (cross-sectional study, CS) and (ii) Study II: Short-term ET (longitudinal study, LS). Due to the lack of established normative values for physical fitness in older populations, we combined cardiorespiratory and strength metrics to categorize participants by fitness level. These groupings were further validated by self-reported physical activity, confirming that the trained group engaged in regular exercise (> 5 years, 2–3 times a week) while the untrained group maintained a sedentary lifestyle. Thus, all participants were divided into terciles, based on the VO_2, peak_ and senior fitness tests score. The groups were defined as: *Sedentary* (*n* = 29), *Moderately trained* (*n* = 29), and *Trained* (*n* = 28). In the frame of CS, we analyzed subgroup of *Sedentary* (*n* = 27, age 72 ± 4 years, BMI 26 ± 5 kg/m^2^,VO_2,peak_ 28.4 ± 5.5 ml/kgFFM/min, senior fitness tests score 6.3 ± 1.0) and *Trained* women (*n* = 25, age 69 ± 4 years, BMI 24.6 ± 2.5 kg/m^2^, VO_2,peak_ 41.0 ± 6.6 ml/kgFFM/min, senior fitness tests score 10.6 ± 1.9).

In LS, 55 women were randomized in two groups, *Placebo* (*n* = 27) and *Calanus* (*n* = 28). *Calanus* group was provided with omega-3 supplementation (Calanus oil) and *Placebo* group was supplemented with sunflower oil. The women were enrolled in physical activity program that consisted of supervised combined aerobic and resistive training 3-times a week for 4 months. In the current study, only *Placebo* group was investigated as Calanus oil supplementation has not been shown to have any additive/different effects on SAT [6] (Suppl. Fig. S1). Twenty-four women completed the LS and agreed on SAT biopsy and twenty-three women were analysed (*n* = 1, not enough of biological material): *Before* and *After ET* (age 70 ± 4 yrs, BMI 27.2 ± 4.1 kg/m^2^, VO_2, peak_ 32.5 ± 4.8 vs. 37.7 ± 5.4 ml/kgFFM/min).

Description of clinical examinations, namely anthropometric and biochemical measurement, blood collection, physical performance testing, biopsy of SAT, and euglycemic-hyperinsulinemic clamp was published before [6]. The number of subjects/samples presented in this paper is specified for the presented method and varies for technical issues.

### Isolation of adipocytes and stromal-vascular fraction from subcutaneous adipose tissue

Adipocytes and stromal vascular-fraction (SVF) were isolated from SAT obtained by needle biopsy. The biopsies were performed before the intervention and 48 h after the last exercise session in the abdominal area, 8 to 10 cm laterally from the umbilicus as described [6]. SAT (1.5–2 g) was cleaned from blood vessels and fibrous material, minced into pieces, and digested in 1.5 volume of collagenase I (300 units/ml, Serva, Germany) for 60 min in a shaking water bath (37 °C). Digested tissue was filtered through a 250 μm strainer to remove undigested scraps, diluted with PBS, and centrifuged at 300 g for 10 min. The upper phase containing mature adipocytes was collected for size determination. A pellet containing SVF was incubated in erythrocyte lysis buffer for 10 min at room temperature and filtered subsequently through 100 μm and 40 μm strainers. Cells were pelleted at 300 g, 10 min, then resuspended in proliferation medium (DMEM/F12, supplemented with 17µM pantothenate, 33µM biotin, 100U/ml penicillin-streptomycin mixture (basal medium), 0.8 µg/ml insulin, 2.5% FBS, 10ng/ml EGF, and 1ng/ml FGF) and plated on 35 mm Petri dish for further cultivation (Fig.2A).

### Adipocyte size determination

Isolated adipocytes were washed twice with PBS, then gently mixed and 50 µL aliquot was mixed with 50 µL 1% BSA/PBS solution/methylene blue. Adipocytes were then transferred to a microscope slide with circular spacers drawn by a DAKO pen and covered with plastic Thermanox #1.5 coverslips. Images were captured by inverted microscope Leica DMI 6000 CS with a 10x objective and monochrome fluorescence camera Leica DFC 350 FX via software Leica LAS AF 2.7.2. For each subject, 3 images were taken (on average 350 adipocytes/picture) and analyzed using freeware software CellProfiler combined with in house macro [6].

### Cell cultures

SVF cells were cultivated in proliferation medium upon 70% confluence, the cells were grown till passage 2 and then cryopreserved in liquid nitrogen. For differentiation experiments, all cell samples were plated in the density of 10,000 cells/cm^2^ in one run and allowed to grow until they were 2-days post-confluent. Then the differentiation was started by adipogenesis induction media (DMEM/F12 basal medium supplemented with 66nM insulin, 1µM dexamethasone, 1nM T3, 10 µg/ml transferrin, 0.25mM IBMX, 1µM rosiglitazone), which was changed after 3 days. After 6 days, cells were switched to the adipogenesis maintenance medium (DMEM/F12 basal medium supplemented with 66nM insulin, 1nM T3, 10 µg/ml transferrin, 0.1µM cortisol) until day 12. The level of differentiation was analyzed by Oil Red O staining and mRNA expression (Fig.2A).

### Oil red O (ORO) staining

Cells were fixed in 10% solution of formaldehyde in aqueous phosphate buffer for 10 min, then washed with 60% isopropanol, and stained with 5.2mM Oil Red O solution (in 60% isopropanol) for 10 min followed by repeated washes with water. The dye was eluted into 100% isopropanol within 15 min. The optical density of the eluate was measured at 500 nm [15].

### Gene expression

Total mRNA was isolated from 120 to 200 mg aliquots of SAT using RNeasy Lipid Tissue Mini Kit (Qiagen, Germany) and QIAzol lysis reagent. RNA concentration was measured using Nanodrop1000 (ThermoFisher Scientific, USA). To remove genomic DNA, DNase I (ThermoFisher Scientific) treatment was applied. RNA was reverse transcribed using SuperScript IV Reverse Transcriptase (ThermoFisher Scientific). For microfluidics, 4 ng of cDNA were preamplified within 18 cycles (CS: TaqMan TM PreAmp Master Mix Kit, ThermoFisher Scientific; LS: SsoAdvanced TM PreAmpSupermix, BIORAD, USA). For the preamplification, 20x TaqMan gene expression assays of all target genes were pooled together and diluted with water to the final concentration 0.2x for each probe. After preamplification, DNA was diluted with dH_2_O 20-times. The qPCR was performed in duplicates on Biomark Real Time qPCR system using 96.96 dynamic array (Fluidigm, USA). A total of three different chips were used. Raw data were recalculated according to several interplate controls and data were normalized to the reference gene TBP. For statistical analysis and presentation 2^–ΔCt^ was used.

### Adipose tissue explants, lipolysis induction

SAT aliquot (0.5–1 g) was washed twice in KRH (Krebs/Ringer phosphate buffer, 10mM HEPES, pH 7.4) with 0.5% FFA free BSA, then incubated at 37 °C in 0.5 ml of the same buffer for one hour (shaking water bath) to remove dead cells and other debris. The tissue was gently dried and divided into 100 mg explants. A 0.5 ml of control (KRH 2% BSA) or lipolytic (KRH with 2% FFA free BSA, 100 nM isoproterenol) media was added per 100 mg of tissue [20]. After 4-hour incubation at 37 °C in water bath, media were collected, aliquoted, and kept frozen (-80 °C) for further analysis.

### Analysis of plasma and media

Plasma concentrations of glucose, insulin, and lipids were determined using standard biochemical methods in certified laboratory (Spadia s.r.o., CZ). Glycerol was measured in media by enzymatic colorimetric kit (Randox, UK).

### Untargeted lipidomics in SAT

Untargeted lipidomics was performed at the Institute of Physiology, the Czech Academy of Science. SAT (20 mg) was extracted by a biphasic solvent system of cold methanol, methyl tert-butyl ether, and water [10]. The liquid chromatography-mass spectrometry analysis consisted of a Vanquish UHPLC System (Thermo Fisher Scientific) coupled to a Q Exactive Plus mass spectrometer (Thermo Fisher Scientific) and UltiMate 3000 RSLC UHPLC system coupled to a QTRAP 5500/SelexION massspectrometer (SCIEX) [10]. LC-MS data were processed through MS-DIAL v. 2.52 and 2.80 software [21]. Lipids were annotated using in-house retention time–m/z library and using MS/MS libraries available from public sources (MassBank, MoNA). Raw data were filtered using blank samples, serial dilution samples, and quality control (QC) pool samples with relative standard deviation (RSD) < 30% and then normalized using LOESS approach by means of QC pool samples for each matrix injected regularly between 10 actual samples. Data were further processed via MetaboAnalyst [10]. Partial least squares–discriminant analysis (PLS-DA) with variable importance in projection (VIP) was performed. Molecules with a VIP score > 1.5 were considered the main variables contributing to the separation of groups in the PLS-DA model and were subsequently included in further interpretation. 

### Proteomic analysis in SAT

Proteomic analysis was performed at the Institute of Physiology, Czech Academy of Sciences [22, 23]. Briefly, pieces of white fat corresponding to approx. 20 mg (wet weight) were delipidated by a modified Matyash method using MTBE (methyl-tert-butyl ether) extraction with MTBE/MeOH/H2O 10:3:2.5 (v/v/v). Insoluble protein pellet was dried, dissolved in 100 mM TEAB (triethylammonium bicarbonate) with SDS (final concentration 1.5%, w/v) and processed according to the SP4 no glass bead protocol [23]. Solubilized samples were reduced with 10 mM TCEP [tris(2-carboxyethyl)phosphine], alkylated with 40 mM CAA (chloroacetamide), and digested with trypsin (Trypsin Gold, MS Grade, Promega V5280) over-night at 37 °C at 1:40 ratio (trypsin: protein). Resulting peptides were desalted on C18 StageTips, dried in Speedvac and dissolved in 0.1% TFA + 2% acetonitrile. About 500 ng of peptide digests were separated on a C18 column using nanoUHPLC (Dionex Ultimate 3000) and analyzed in a DIA mode on an Orbitrap Exploris 480 mass spectrometer equipped with a FAIMS unit. Thermo raw files were processed by the directDIA mode and visualized in Spectronaut 19.2 (Biognosys) software using the default settings with Precursor and Protein Q-value and PEP cutoff set at 0.01. Search was performed against the human reference proteome UP000005640_9606.fasta (UniProt release 2024_01). Protein groups quantities (PG.Quantity, MS2 level) from the Protein report generated by the Spectronaut were evaluated.

### Statistical analysis

Statistical analysis was performed using GraphPad Prism 10.3.1 for Windows (LaJolla, USA). All datasets were tested for normality or lognormality using a battery of tests (Shapiro-Wilk, D’Agostino & Pearson, Anderson-Darling, Kolmogorov-Smirnov). Data are presented as means ± SEM, medians ± IQR (mRNA expression) or min to max (lipidomic and proteomic data). Differences between the *Trained* and *Sedentary* women in CS were analyzed by single or multiple Mann-Whitney rank test. Differences between the responses *Before* and A*fter* ET in LS or matched *Sedentary* and *Trained* based on FM in CS were analyzed by single or multiple Wilcoxon test. Correlations were expressed as Pearson’s correlation coefficient. The level of significance was set up at *p* < 0.05 and FDR < 0.05.

## Results

### Physical activity is associated with lower hypertrophy of SAT

We first examined the effects of training on obesity/adiposity parameters, cardiorespiratory fitness, and insulin sensitivity (IS). As expected, long-term *Trained* women had lower adiposity compared to *Sedentary* women (*Trained* (*n* = 25) vs. *Sedentary* (*n* = 24): weight 63.4 ± 7.2 kg vs. 68.7 ± 7.2 kg, *p* = 0.008; FM 36.5 ± 4.3% vs. 40.6 ± 5.0%, *p* = 0.004; waist circumference 79.1 ± 6.9 cm vs. 85.3 ± 7.1 cm, *p* = 0.002). The *Trained* women also demonstrated better cardiorespiratory fitness and IS (VO_2, peak_ 28.4 ± 5.5 vs. 41.0 ± 6.6 ml/kgFFM/min, *p* < 0.0001; M_kg_ 6.8 ± 2.1 vs. 5.9 ± 2.1 mg/kg/min, *p* = 0.016). To assess whether these differences were independent of fat mass, we selected two subgroups of *Sedentary* and *Trained* women matched one-to-one for FM (*n* = 10 vs. 10). In these matched subgroups, the difference in cardiorespiratory fitness remained significant (*p* = 0.002), indicating that this parameter is not dependent on adiposity alone in older women. However, IS (M_kg_) did not differ after matching, suggesting a dependence on adiposity.

Short-term ET did not induce change in body weight, but the amount of FM was significantly reduced (*Before* vs. *After ET* (*n* = 24): 38.8 ± 5.2 vs. 37.4 ± 5.1%, *p* < 0.0001). Short term ET also improved cardiorespiratory fitness, but not IS (*Before* vs. *After ET* (*n* = 24): VO_2, peak_ 32.5 ± 4.8 vs. 37.7 ± 5.4 ml/kgFFM/min, *p* = 0.0002, M_kg_ 5.3 ± 1.9 vs. 5.7 ± 2.3 mg/kg/min, *p* = 0.182) [6].

**Fig. 1:**
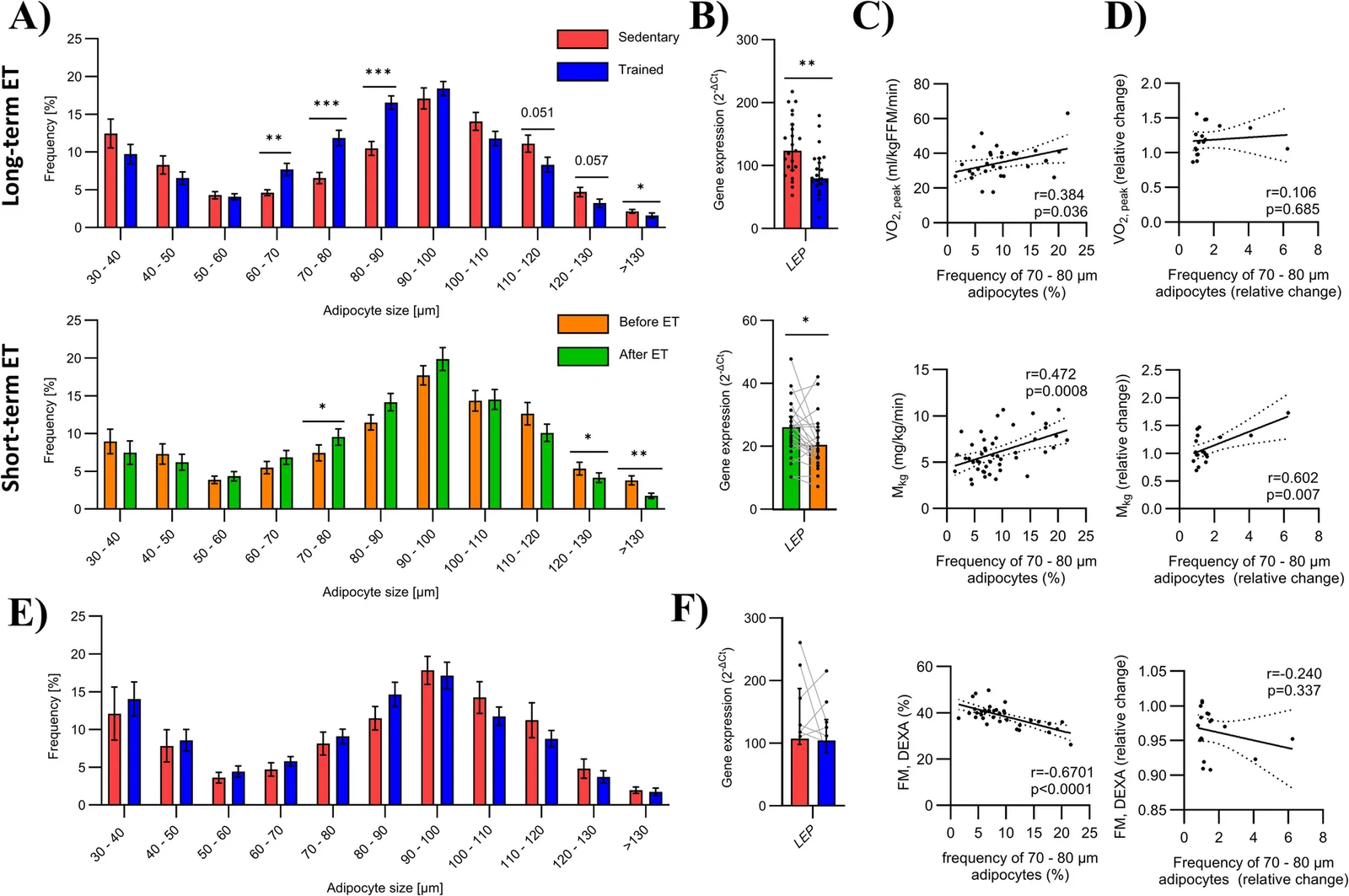
Adipocyte size in SAT. **A** Relative frequency of adipocytes in defined size range (long-term ET: bin 10 μm, Sedentary vs. Trained, *n* = 24 vs. 25; short-term ET: bin 10 μm, Before vs. After, *n* = 19; technical triplicate). **B** Expression level of leptin mRNA in SAT (long-term ET: Sedentary vs. Trained, *n* = 23 vs. 21; short-term ET: Before vs. After, *n* = 23; technical duplicate). Correlations of adipocyte size frequency and adiposity and insulin sensitivity parameters (Pearson‘s correlation coefficient) (**C**) in long- and (**D**) short-term ET. **E** Relative frequency of adipocytes in defined size range (Sedentary vs. Trained, *n* = 10; matched by FM, bin 10 μm; technical triplicate). **F** Gene expression level of leptin mRNA in SAT (Sedentary vs. Trained, *n* = 7; matched by FM, technical duplicate). Data are presented as mean ± SEM (A, E) and median ± IQR (B, F). Mann-Whitney rank test (long-term ET) or Wilcoxon test (short-term, long-term ET matched by FM) were performed, p-value: *p < 0.05, **p < 0.01, ***p < 0.001. ET, exercise training; SAT, subcutaneous adipose tissue

To assess the extent of SAT hypertrophy, we analyzed the size of isolated adipocytes and the gene expression of leptin in SAT [24]. The mean adipocyte size did not differ significantly between groups in either part of the study (*Sedentary* (*n* = 19) vs. *Trained* (*n* = 25): 70.2 ± 17.4 vs. 74.1 ± 12.8 μm, *p* = 0.313; *Before* vs. *After* ET (*n* = 23): 74.7 ± 13.2 vs. 75.4 ± 14.8 μm, *p* = 0.899 [6]). However, we observed a lower proportion of hypertrophic adipocytes (≥ 110 μm) in *Trained* compared to *Sedentary* women, and a decrease in these larger adipocytes (> 120 μm) after short-term ET (Fig. 1A). Additionally, there was a higher proportion of medium-sized adipocytes (60–90 μm) in *Trained* women and an increase in 70–80 μm adipocytes after short-term ET (Fig. 1A). Consistent with these findings, leptin mRNA was significantly lower in both groups of trained women (Fig. 1B).

The relative number of medium-sized adipocytes correlated positively with cardiorespiratory fitness (VO_2, peak_), IS (M_kg_) and negatively with FM in both studies (Fig. 1C, D). These results suggest that lower adipocyte hypertrophy in *Trained* women may contribute to higher IS and cardiorespiratory fitness in this group. However, the comparison of FM matched subgroups showed no significant difference in adipocyte distribution and leptin gene expression (Fig. 1E, F), which indicates that the observed reduction in SAT hypertrophy is primarily a consequence of reduction in participants’ FM/adiposity. To mitigate the effect of adiposity, we performed also multiple regression analysis, which confirms the dependence of adipocyte size on adiposity (FM).

### Physical activity does not increase adipogenic potential

**Fig. 2:**
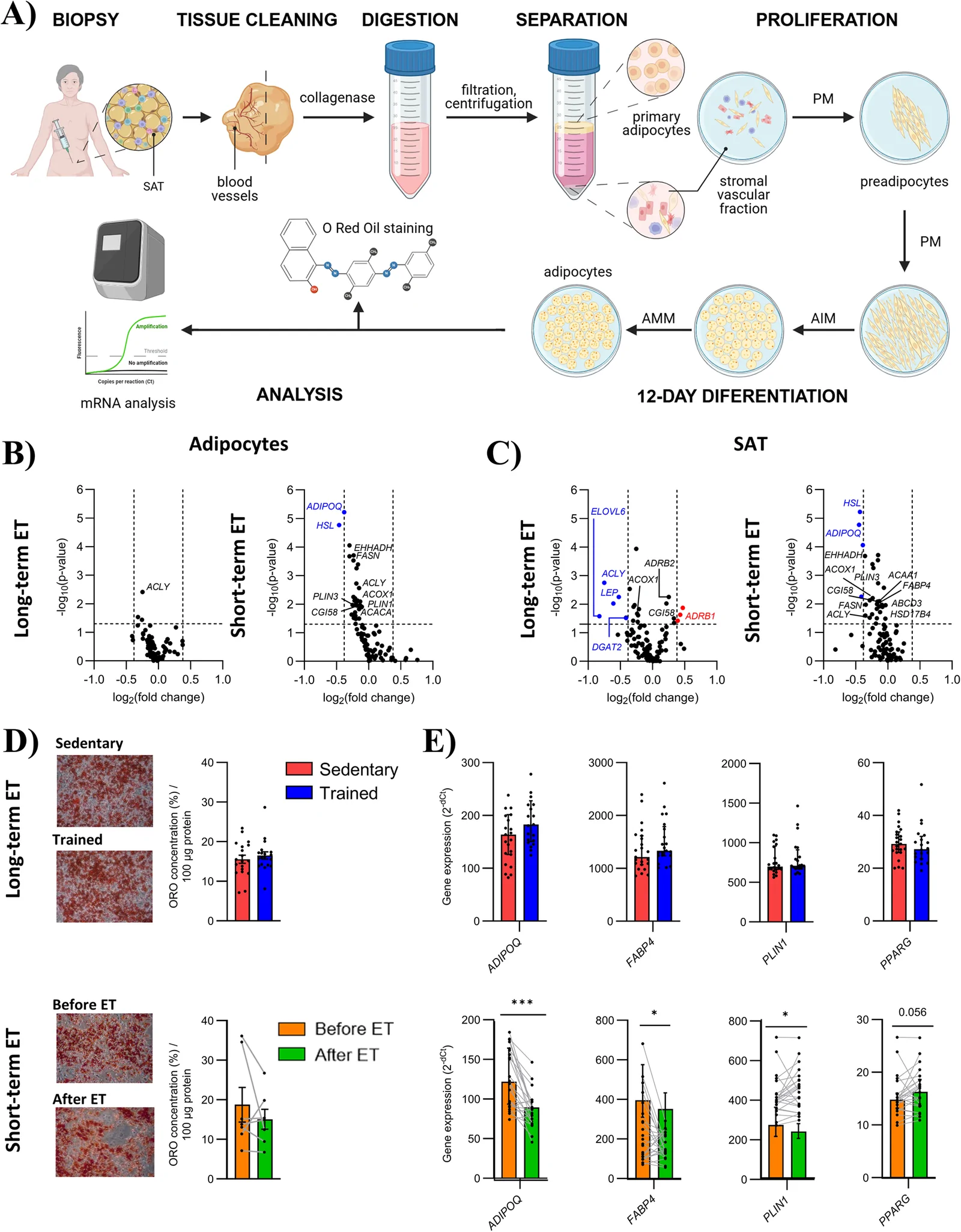
Gene expression and adipogenic capacity in SAT. **A** Sample processing scheme and cell culture set up (created by BioRender.com). Volcano plot of (**B**) adipocyte and (**C**) SAT gene expression (long-term ET: Sedentary vs. Trained, *n* = 15 vs. 18 (adipocytes), *n* = 27 vs. 24 (SAT); short-term ET: Before vs. After, *n* = 7 (adipocytes), *n* = 23 (SAT); technical duplicates). **D** Differentiated adipocytes in long- and short-term ET: representative photos of cells stained with Oil Red O; O Red Oil concentration normalized to protein content (long-term ET: Sedentary vs. Trained, *n* = 19 vs. 19; short-term ET: Before vs. After, *n* = 7; technical duplicates). **E** Gene expression level of adiponegic markers in SAT (long-term ET: Sedentary vs. Trained, *n* = 27 vs. 24; short-term ET: Before vs. After, *n* = 23, technical duplicates). Data are presented as mean ± SEM (**D**), median ± IQR (**E**). Mann-Whitney rank test (long-term ET) or Wilcoxon test (short-term ET) were performed; gene expression: p-value: *p < 0.05, **p < 0.01, ***p < 0.001. AIM, adipogenesis induction medium; AMM, adipogenesis maintenance medium; ET, exercise training; PM, proliferation medium; SAT, subcutaneous adipose tissue

Higher adiposity and hypertrophy of adipose tissue may be closely related to lower adipogenic capacity of AT, which is also negatively associated with metabolic health [15, 25]. During adipogenesis, new lipids are formed and accumulated; therefore, the total lipid content in the sample was assessed using the Oil Red O method combined with gene expression analysis. We analyzed mRNA expression using a 96.96 Dynamic Array in adipocytes and SAT, covering a broad panel of selected metabolic markers with a particular focus on adipogenic capacity and lipid metabolism (including lipolysis, de novo lipogenesis, and peroxisomal β-oxidation). Specifically, 80 genes were measured in adipocytes and 103 genes in SAT in the long-term ET group, while 90 genes were measured in adipocytes and 91 genes in SAT in the short-term ET group (Fig. 2B, C).

We found no difference in lipid accumulation in either long- or short-term ET (Fig. 2D). Considering the adipogenic markers (*ADIPOQ*,* FABP4*,* PLIN1*, and *PPARG*) we found no difference between *Sedentary* and *Trained* women in mRNA, either in adipocytes or in whole SAT (Fig. 2B and E). Following short-term ET, however, there was a significant reduction in *ADIPOQ. FABP4* and *PLIN1* were also decreased, in both adipocytes and SAT, while there was a trend toward higher *PPARG* expression (*p* = 0.057) (Fig. 2B and E).

Finally, when we compared FM matched subgroups from long-term ET, no differences were found between the groups (data not shown). Together, these data do not suggest any changes in ET-related adipogenic capacity in older women.

### Lipidomic analysis

**Fig. 3:**
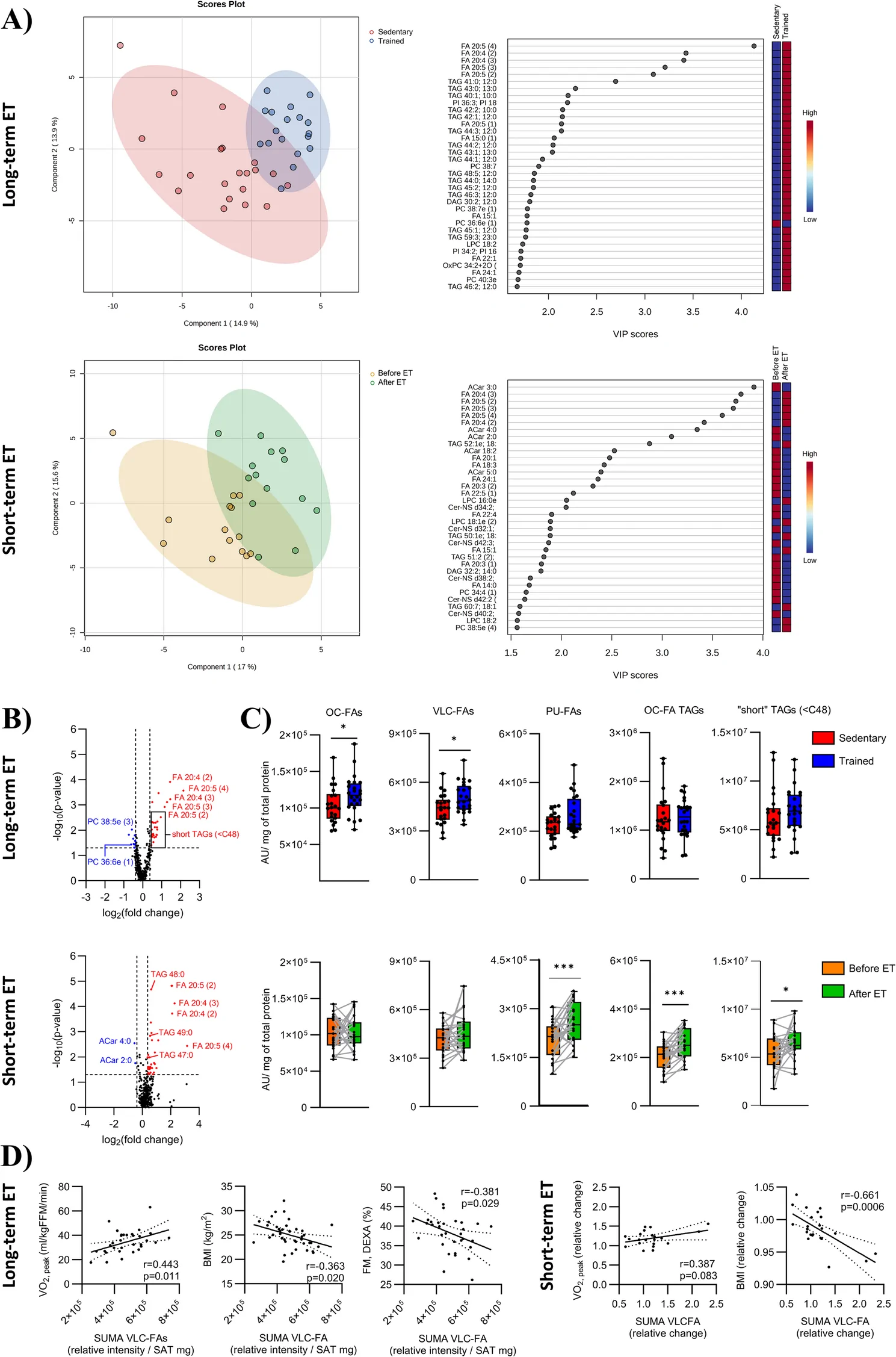
Lipidomic analysis in SAT. **A** PLS-DA and top 35 lipid molecules with VIP score > 1.5 (long-term ET: Sedentary vs. Trained, *n* = 21 vs. 18; short-term ET: Before vs. After, *n* = 14, technical monoplicate). **B** Volcano plot of lipid molecules (long-term ET: fold change: Trained/Sedentary, *n* = 18 vs. 21; short-term ET: fold change After/Before, *n* = 14). **C** Selected lipid classes (long-term ET: Sedentary vs. Trained, *n* = 21 vs. 18; short-term ET: Before vs. After: *n* = 14, technical monoplicate). **D** Correlations of suma VLC-FA, adiposity and cardiorespiratory fitness parameteres (Pearson‘s correlation coefficient). Data are presented as min to max (**C**), Mann-Whitney rank test (long-term ET) or Wilcoxon test (short-term ET) were performed: p-value: *p < 0.05, **p < 0.01, ***p < 0.001. ET, exercise training; PLS-DA, partial least square – discriminant analysis; SAT, subcutaneous adipose tissue; VIP, varible importance in projection

As our previous findings showed important effects of ET on several lipid mediators, particularly increase in PAHSA in response to short-term ET [10], we further analyzed our lipidomic data in long- and short-term ET. A total of 415 lipid molecules in both long-term and short-term ET were analyzed. PLS-DA analysis and VIP scores identified the most abundant lipid classes. In long-term ET, of 51 lipid molecules with VIP > 1.5 included mainly short TAGs (39%), FFAs (24%) and VLC-FA TAGs (24%) (Fig. 3A). All these lipid classes were lower in *Trained* women compared to *Sedentary* group (Fig. 3B). Furthermore, in the short-term ET, 58 molecules were identified with a VIP score > 1.5, including FFAs, short TAGs, and PUFA TAGs (16% per group) (Fig. 3A). All of FFAs with VIP score > 1.5 were up-regulated *After* ET and most of the short TAGs as well (Fig. 3B).

We found that the length of physical activity was decisive of lipidome alterations. While adaptation resulted in higher content of odd-chain (OC-FAs) and saturated/monosaturated very-long chain (> 20 C chain) fatty acids (VLC-FAs) in long-term trained women, the effect of short-term ET increased content of polyunsaturated FA (PUFA), triacylglycerols with short/saturated fatty acids (< C48, “short” TAGs) and with OC-FAs (OC-FA TAGs) in SAT (Fig. 3C, Suppl. Material 2).

To reveal a possible association of these lipid molecules with whole body metabolic health, we correlated these lipid classes with body composition, IS and VO_2, peak_. We observed that the sum of VLC-FA in SAT positively correlated with the cardiorespiratory fitness parameter (VO_2, peak_) and negatively with adiposity parameters in group of long-term *Trained* and *Sedentary* women and also in the changes during short-term ET (Fig. 3D). Based on the multiple regression analysis with fat mass and BMI as covariate we suggest that the correlation of VO_2, peak_ with VLC-FA is independent on fat mass accumulation (β = 3.177·10^− 5^, *p* = 0.047, F[1, 28] = 4.32, VIF = 1.146).

### Lipolytic response depends on the duration of ET

**Fig. 4:**
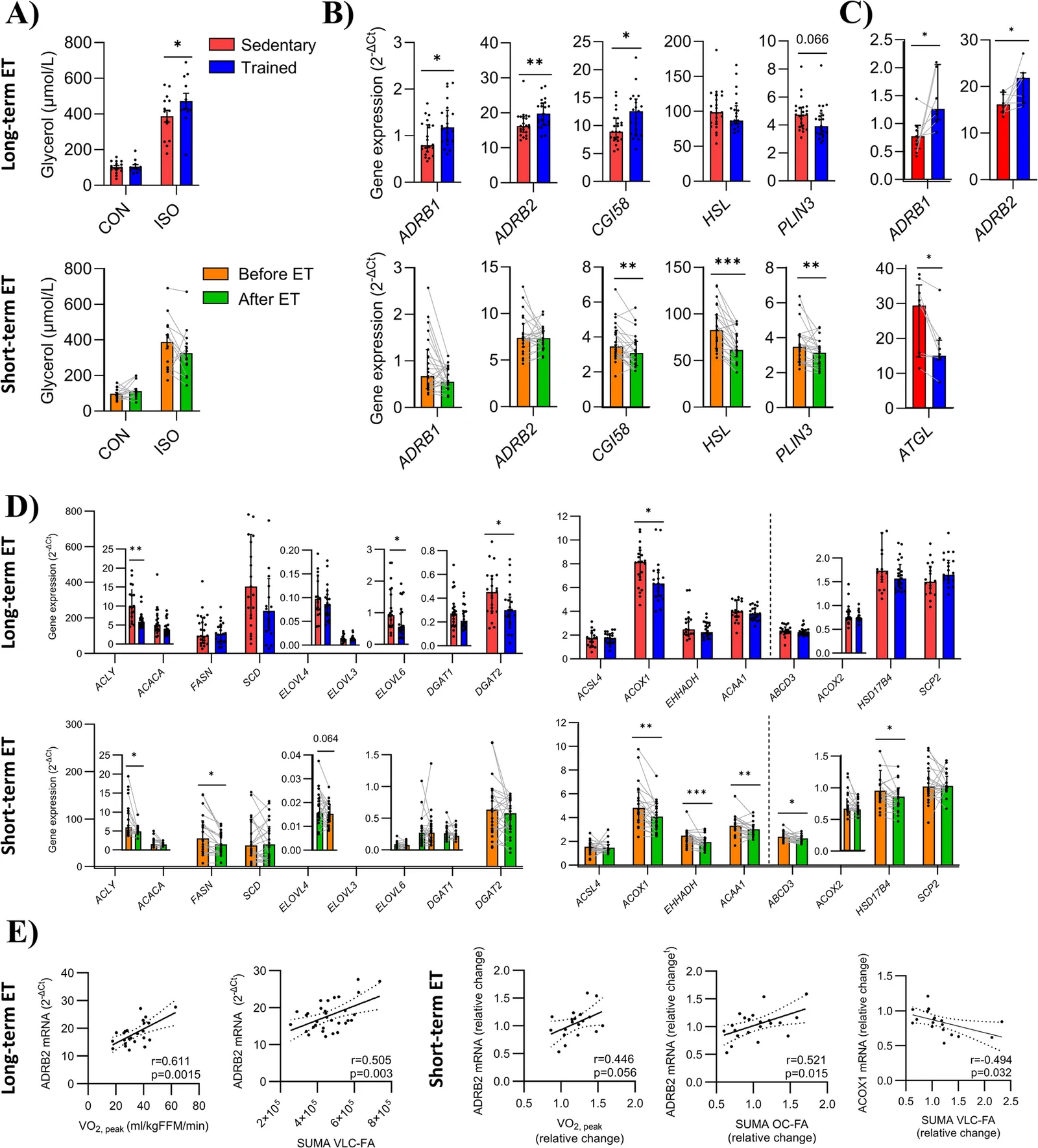
Lipid metabolism in SAT. **A** Glycerol release in SAT explants (long-term ET: Sedentary vs. Trained, *n* = 14 vs. 11; short-term ET: Before vs. After, *n* = 14, technical duplicates). **B** Gene expression level of lipolytic markers (long-term ET: Sedentary vs. Trained, *n* = 27 vs. 24; short-term ET: Before vs. After, *n* = 23, technical duplicates). **C** FM-matched gene expression level of lipolytic markers (long-term ET: Sedentary vs. Trained, *n* = 10; matched by FM, bin 10 μm, technical duplicate). **D** Gene expression level of DNL and peroxisomal β-oxidation markers (long-term ET: Sedentary vs. Trained, *n* = 27 vs. 24; short-term ET: Before vs. After, *n* = 23; technical duplicates). **E** Correlations of ADRB2 mRNA expression, suma of VLC-FA, OC-FA and cardiorespiratory fitness parameter (Pearson‘s correlation coefficient). Data are presented as mean ± SEM (**A**), median ± IQR (**B**, **C**, **D**). Mann-Whitney rank test (long-term ET) or Wilcoxon test (short-term ET) were performed, p-value: *p < 0.05, **p < 0.01, ***p < 0.001. CON, control; ET, exercise training; ISO, 0.1µM isoproterenol, SAT, subcutaneous adipose tissue

To determine the rate of lipolysis we analyzed the amount of glycerol released ex vivo from SAT explants, in non-stimulated (basal) and isoproterenol-stimulated conditions. The higher lipolytic response to isoproterenol was present in the long-term *Trained* group, whereas there was no change in lipolytic response after short-term ET (Fig. 4A).

Seven markers of lipolysis (*ADBR1*, *ADBR2*, *ATGL*, *CGI58*, *HSL*, *PLIN2* and *PLIN3*) were measured in differentiated adipocytes and in SAT. In long-term ET, there was no change between lipolytic markers in adipocytes (Fig. 2B), whereas in SAT, *ADRB1*,* ADRB2*, and *CGI58* mRNA were higher, and *PLIN3* had trend to be lower in long-term *Trained* women to compare *Sedentary* women. *ATGL*,* HSL* and *PLIN2 levels* were not different between the groups (Figs. 2C and 4B). Furthermore, comparison of matched subgroups by FM revealed higher mRNA of *ADRB1*, *ADRB2* and lower *ATGL* in long-term *Trained* women (Fig. 4C). In response to the short-term ET, *HSL* was down-regulated *After* ET, whereas *CGI58* and *PLIN3* gene expression was decreased in adipocytes (Fig. 2B). *CGI58*, *HSL* and *PLIN3 mRNA expression*, were down-regulated. *ADRB1*,* 2*,* ATGL* and *PLIN2* mRNA expressions were not changed in SAT (Figs. 2C and 4B).

Thus, taken together, short-term ET appears to reduce the mRNA expression of lipolytic pathway in SAT but the longer-term physical activity increases this expression. Interestingly, mRNA expression of adrenergic receptors (*ADRB2*) correlated positively to VLC-FAs in long-term *Trained* and *Sedentary* women, and to VO_2, peak_ in long- and short- term ET (Fig. 4E). To reveal the effect of adiposity on the correlations in long-term ET, we performed multiple regression analyses as well. Based on this analysis we may suggest that VO_2, peak_ is significantly dependent on *ADRB2* mRNA expression independently on FM (*ADRB2*: F[1,21] = 7.601, β = 1.238, *p* = 0.012; VIF = 1.127).

### Changes in *de novo* lipogenesis and peroxisomal metabolism during short- and long-term ET

Because VLC-FAs and branched-chain fatty acids (BC-FAs) (several OC-FAs belong to BC-FAs) are metabolized by peroxisomes, and “short” TAGs are associated with *de novo* lipogenesis (DNL) [26] we explored these pathways also on the mRNA expression level of their markers/enzymes.

DNL was investigated by assessing the mRNA levels of 9 enzymes (*ACLY*, *ACACA*, *FASN*, *SCD*, *ELOVL3*,* ELOVL4*,* ELOVL6*, *DGAT1* and *DGAT2*) and focused on peroxisomal β-oxidation of VLC-FAs (*ACSL4*,* ACOX1*,* EHHADH*,* ACAA1*) and BC-FAs (*ABCD3*,* ACOX2*,* HSD17B4* and *SCP2*).

In long-term ET, only *ACLY* expression in adipocytes was lower in *Trained* women compared to *Sedentary* (Fig. 2B). On the other hand, in SAT, the mRNA levels of four genes in the DNL pathway (*ACLY*,* DGAT2*,* ELOVL4*, and *ELOVL6*) and one gene involved in VLC-FA oxidation (*ACOX1*) were lower in long-term *Trained* women compared to *Sedentary* women (Figs. 2B and C and 4D). In response to short-term ET, three lipogenic genes (*ACLY*,* ACACA* and *FASN*) and two genes involved in peroxisomal β-oxidation of VLC-FA (*ACOX1*,* EHHADH*) were decreased in adipocytes (Fig. 2B). In addition, we observed similar results in the SAT, though on a different range, DNL (*ACLY*, *FASN*) and peroxisomal β-oxidation (*ACOX1*, *EHHADH*, *ACAA1*, *ABCD3*, *HSD17B4*) were down-regulated (Fig. 4D).

No significant correlation among the exploratory variables and IS was found. We only observed negative correlations between changes in *ACOX1* mRNA and sum of VLC-FAs (*r*=-0.494, *p* = 0.032) during short-term ET (Fig. 4E).

### Proteomic analysis in SAT

Finaly we performed also proteomic analysis in selected subgroups of women who exhibited the greatest differences in VO_2, peak_ parameter (CS) and/or the highest improvement in VO_2, peak_ following the training intervention (CS: Sedentary (*n* = 9) vs. Trained (*n* = 8): VO_2, peak_ 29.1 ± 6.0 vs. 38.5 ± 5.5 ml/kgFFM/min, *p* = 0.02; LS (*n* = 14): Before vs. After ET VO_2, peak_: 30.4 ± 7.8 vs. 38.4 ± 4.7 ml/kgFFM/min, *p* = 0.0002).

**Fig. 5:**
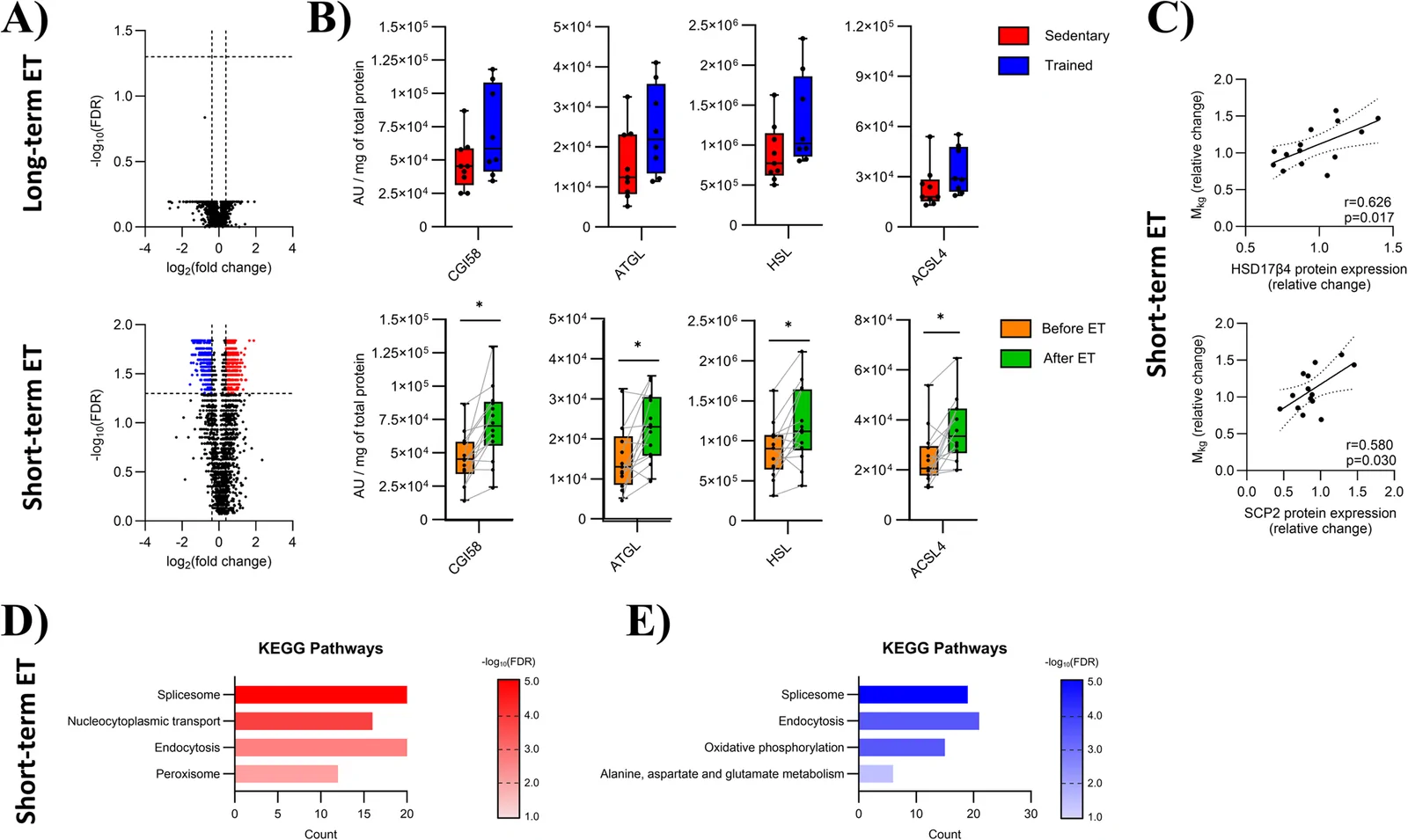
Proteomic and enrichment analysis in SAT. **A** Volcano plot of protein expression (long-term ET: Sedentary vs. Trained, *n* = 9 vs. 8; short-term ET, Before vs. After, *n* = 14; FDR < 0.05, fold change > 30%; technical monoplicate). **B** Protein expression of selected markers ((long-term ET: Sedentary vs. Trained, *n* = 9 vs. 8; short-term ET, Before vs. After, *n* = 14; *FDR < 0.05). **C** Correlations of HSD17B4, SCP2 and IS parameters (Pearson‘s correlation coefficient). Enrichment analysis of (**D**) up-regulated and (**E**) down-regulated proteins (FDR < 0.1). ET, exercise training; FDR, false discovery rate; SAT, subcutaneous adipose tissue

A total of 7,141 proteins in SAT were identified, of which 4,756 were analyzed in long-term ET and 4,951 in short-term ET after exclusion of proteins with more than 50% missing values. In long-term ET, *Trained* women did not differ significantly compared to *Sedentary* women (FDR < 0.05) (Fig. 5A). In short-term ET, 993 proteins were changed during ET (536 up-regulated, 457 down-regulated, FDR < 0.05). Considering selected metabolic pathways only CGI58, ATGL, HSL and ACSL4 protein expression was upregulated *After* ET, while in long-term ET, there were similar trends observed without statistical significance (Fig. 5A, B). The levels of other proteins in the selected metabolic pathways (lipolysis, DNL, and peroxisomal metabolism) were not changed in association with long- and short-term ET.

Subsequently we performed enrichment analysis (https://maayanlab.cloud/Enrichr/) based on differentially expressed proteins (611 down-regulated, 808 up-regulated, FDR < 0.1) in short-term ET. Based on Reactome and KEGG libraries, the pathways significantly affected by ET included “spliceosome”, “peroxisome metabolism”, “metabolism of proteins”, RNA metabolism, endocytosis and oxidative metabolism (Fig. 5D, E; Suppl. Material 3 and 4).

## Discussion

Adipocyte size, i.e., the degree of AT hypertrophy in SAT is one of the measures of AT dysfunction we analyzed. The lower percentage of hypertrophic adipocytes and the higher amount of smaller/medium adipocytes in more trained individuals suggests that physical activity is associated with better AT characteristics. Nevertheless, the loss of this effect in subgroups matched by FM indicates that reduced SAT hypertrophy is predominantly a result of the decline in FM, but not as an effect of exercise itself. These results align with previous studies on the effect of ET in young/middle age individuals with obesity [8, 9, 27], indicating that the missing impact of ET on adipocyte hypertrophy is a widespread phenomenon not specific to age or gender. Accordingly, we have not found a clear improvement of intrinsic adipogenic capacity of adipose precursor in response to ET, which could be responsible for limiting AT hypertrophy [15, 25]. At the levels of gene and protein expression, we observed slight increase in mRNA levels of *PPARγ*, a major adipogenic transcription factor, but the other markers were not changed or were decreased. Similar results have been published in obese men after 6-months of training [11], where an increase in *PPARγ* expression was observed in SAT and muscle. On the other hand, there was no change in *PPARγ* expression after 12-week and more than 2-year training in individuals with overweight and obesity [7, 8, 27]. Thus the effect of exercise on adipogenesis is still questionable. Based on our results we assume that the intrinsic adipogenic capacity of SAT is not markedly changed, however, we cannot exclude that in *vivo* the adipogenesis may be partially improved, possibly due to changes in microenvironment (vascularization, ECM changes) or endocrine signals [6, 7, 9].

We also focused on lipolytic activity as an important parameter of SAT functionality. In long-term *Trained* women, we observed higher response of SAT to catecholamines and at the same time, higher levels of adrenergic receptors and CGI58, an ATGL activator. Importantly, ADRB1 and ADRB2 remained higher in *Trained* women even in subgroups matched by FM, demonstrating the impact of long-term physical activity on adrenergic stimulation regardless of adiposity. On the other hand, after short-term ET, we did not observe any change in the lipolytic response (glycerol release), despite increased protein levels of the two main lipase proteins (HSL, ATGL) and CGI58. Moreover, protein expression differed significantly from mRNA levels, which were reduced after short-term ET. These results differ slightly from studies in mice and obese individuals, in which an increase in catecholamine-stimulated lipolysis and HSL activity was observed after 12 weeks of ET [8, 28–30]. Based on our data, it appears that in older women, the effect of physical activity on increased adrenergic stimulation is somewhat delayed and only becomes apparent after prolonged training. Notably, aging itself changes the interplay between mRNA and protein expression [31]. Within the cardiovascular system, it has previously been confirmed that physical activity can prevent the desensitization of the adrenergic system during aging and thus positively regulate blood pressure [32]. The observation that ADRB2 expression correlated with cardiorespiratory fitness independently of adiposity highlights this pathway as a potential mediator of exercise-induced adaptations, warranting further investigation.

Considering protein and lipid levels, our results suggest that exercise affects the metabolism of VLC-FAs and OC-FAs. After short-term ET, VLC-FAs and OC-FAs were increased in TAGs, while free VLC-FAs and OC-FAs were higher in long-term *Trained* women. Increased levels of free fatty acids may result from reduced β-oxidation, increased synthesis, or increased release from TAGs. The positive correlation between free VLC-FAs and *ADRB2* receptor expression suggests that this increase may be related to higher lipolytic activity in long-term *Trained* women. Elevated levels of acyl-CoA synthetase long chain family member 4 (ACSL4), which converts free long-chain fatty acids into fatty acyl-CoA esters, further support increased metabolic activation of VLC-FAs after ET. Notably, VLC-FAs, PUFAs, and BC-FAs - especially their thioesters with CoA - are important ligands for peroxisome proliferator-activated receptors (PPARα and PPARγ) [33], which enhance mitochondrial and peroxisomal metabolism. VLC-FAs and PUFAs are also key precursors for the synthesis of sphingolipids and glycerophospholipids, which are essential for mitochondrial membrane formation [34]. Therefore, we may speculate that elevated levels of these fatty acids in response to ET contribute to membrane remodeling or PPAR activation. The correlation with cardiorespiratory fitness (VO_2, peak_) further indicates that these outcomes are possibly related to the level of physical activity.

Based on the general proteomic and enrichment analysis the metabolism of proteins and peroxisomes were enriched after ET, together RNA metabolism and splicing. Notably, in recent study the alternative splicing was identified as a major mediator of the responses to ET [35]. From the identified pathways HSD17β4, and SCP2 proteins were found to correlate with insulin sensitivity. HSD17β4, 17β-hydroxysteroid dehydrogenase type 4, is responsible for peroxisomal β-oxidation of fatty acids, and also to docosahexaenoic acid generation, which is essential for membrane composition and anti-inflammatory mediators formation [36]. This finding again points to the membrane remodeling as mentioned before.

This study has several strengths and limitations. Combined assessment of SAT adipogenic capacity, gene expression, lipidomic and proteomic data together with detailed phenotypization of subjects are unique across the other studies not only in older women but generally in human individuals, as well as the comparison of long-term and short-term physical activity themselves. The limitations include that adipogenic capacity was studied in the presence of 1µM rosiglitazone, a strong inducer of adipogenesis, thus subtle differences in adipogenic capacity might be uncovered. Further, an important dichotomy between mRNA and protein levels was present in both parts of the study. While mRNA expression of several metabolic pathways was reduced, protein levels showed no change or a trend toward increase. A similar discrepancy between transcriptomic and proteomic profiles was reported in another study involving individuals with overweight and obesity [37]. The authors suggested that decreases in the expression of metabolic genes (e.g., *ACACA*,* IRS1*,* PPARG*,* SREBF1*,* PNPLA3*,* FASN*) may be influenced by circadian rhythms in mRNA expression, and that long-term adaptation may normalize the amplitude of these circadian fluctuations [37]. However, we have no other supporting data for this interpretation. In addition, given the exploratory nature of the study and the sample size, all the results should be interpreted as indicative.

## Conclusion

In conclusion, we show that in older women, both long-term physical activity and short-term ET are associated with reduced adipocyte hypertrophy, a benefit primarily driven by lower overall adiposity rather than changes in adipogenic capacity. Notably, long-term trained individuals exhibited higher levels of SAT VLC-FAs, which correlated with cardiorespiratory fitness and ADRB2 expression. These findings generate new hypotheses regarding the role of specific VLC-FA species in exercise-induced metabolic adaptations. Further research into the metabolic fates of these lipids and their potential to modulate ADRB2 signaling could identify novel therapeutic targets to mimic the metabolic benefits of exercise in populations with limited mobility. 

## Supplementary Information

Supplementary Material 1.

Supplementary Material 2.

Supplementary Material 3.

Supplementary Material 4.

## Data Availability

The datasets and materials generated and/or analysed during the current study are available from the corresponding author on reasonable request. Supplementary Material is available on DOI 10.5281/zenodo.17648081.

## Abbreviations

BC-FA: Branched-chain fatty acid
BMI: Body mass index
BSA: Bovine serum albumin
CS: Cross-sectional study
DNL: *De novo* lipogenesis
ET: Exercise training
FM: Fat mass
FFM: Fat free mass
IS: Insulin sensitivity
LS: Longitudinal study
OC-FA: Odd-chain fatty acid
ORO: O red Oil
PUFA: Polyunsaturated fatty acid
SAT: Subcutaneous adipose tissue
SVF: Stromal-vascular fraction
TAG: Triacylglycerol
VLC-FA: Very long-chain fatty acid

## Declarations

The study is part of the clinical trial registered on December 18, 2017 as EXODYA (Effect of EXercise training and Omega-3 fatty acids on metabolic health and DYsfunction of Adipose tissue in older, No: NCT03386461, clinicaltrials.org).
