# Supplementary material for: Exercise training is associated with changes in adipocyte size and lipid metabolism in older women: cross-sectional and longitudinal study
Source: BMC Geriatr. 2026 Jun 23;26:1132. doi: 10.1186/s12877-026-07752-9 (PMC13543450; doi:10.1186/s12877-026-07752-9)
Supplement: Supplementary file 1 [file 12877_2026_7752_MOESM2_ESM.docx]

**Suppl. Tab. S1. List of lipid classes.**

**OC-FA**

FA 15:0 (1)

FA 15:0 (2)

FA 15:1

FA 17:0

FA 17:1

FA 19:0

FA 19:1

**PU-FA**

FA 20:4 (1)

FA 20:4 (2)

FA 20:4 (3)

FA 20:5 (1)

FA 20:5 (2)

FA 20:5 (3)

FA 20:5 (4)

FA 22:4

FA 22:5 (1)

FA 22:5 (2)

FA 22:6

**VLC-FA**

FA 20:0 (1)

FA 20:0 (2)

FA 20:1

FA 20:2

FA 20:3 (1)

FA 20:3 (2)

FA 20:4 (1)

FA 20:4 (2)

FA 20:4 (3)

FA 20:5 (1)

FA 20:5 (2)

FA 20:5 (3)

FA 20:5 (4)

FA 22:0

FA 22:1

FA 22:4

FA 22:5 (1)

FA 22:5 (2)

FA 22:6

FA 24:1

**OC-FA TAGs**

TAG 41:0; 12:0-14:0-15:0

TAG 43:0; 13:0-14:0-16:0

TAG 43:1; 13:0-14:0-16:1

TAG 45:0; 14:0-15:0-16:0

TAG 45:1; 12:0-15:0-18:1

TAG 45:2; 12:0-15:0-18:2

TAG 47:0; 14:0-16:0-17:0

TAG 47:1; 14:0-15:0-18:1

TAG 47:2; 14:0-15:0-18:2

TAG 49:0; 15:0-16:0-18:0

TAG 49:1; 15:0-16:0-18:1

TAG 49:2; 15:0-16:1-18:1

TAG 49:3; 15:0-16:1-18:2

TAG 51:1; 16:0-17:0-18:1

TAG 51:2 (1); 16:0-17:1-18:1

TAG 51:2 (2); 16:0-17:1-18:1

TAG 51:3; 15:0-18:1-18:2

TAG 51:4; 16:1-17:1-18:2

TAG 53:1; 17:0-18:0-18:1

TAG 53:2; 17:0-18:1-18:1

TAG 53:3; 17:0-18:1-18:2

TAG 53:4; 17:1-18:1-18:2

TAG 53:5; 17:0-18:2-18:3

TAG 55:2; 18:0-18:1-19:1

TAG 55:3; 18:1-18:1-19:1

TAG 55:4; 18:1-19:1-18:2

TAG 57:1; 16:0-25:0-16:1

TAG 57:2; 23:0-16:1-18:1

TAG 57:3; 18:1-19:1-20:1

TAG 59:1; 16:0-25:0-18:1

TAG 59:2 (1); 23:0-18:1-18:1

TAG 59:2 (2); 25:0-16:1-18:1

TAG 59:3; 23:0-18:1-18:2

TAG 61:2; 27:0-16:1-18:1

**"short" TAGs (<C48)**

TAG 38:0; 12:0-12:0-14:0

TAG 40:0; 10:0-14:0-16:0

TAG 40:1; 10:0-12:0-18:1

TAG 41:0; 12:0-14:0-15:0

TAG 42:0; 12:0-14:0-16:0

TAG 42:1; 12:0-14:0-16:1

TAG 42:2; 10:0-14:0-18:2

TAG 43:0; 13:0-14:0-16:0

TAG 43:1; 13:0-14:0-16:1

TAG 44:0; 14:0-14:0-16:0

TAG 44:1; 12:0-14:0-18:1

TAG 44:2; 12:0-14:0-18:2

TAG 44:3; 12:0-14:0-18:3

TAG 45:0; 14:0-15:0-16:0

TAG 45:1; 12:0-15:0-18:1

TAG 45:2; 12:0-15:0-18:2

TAG 46:0; 14:0-16:0-16:0

TAG 46:1; 12:0-16:0-18:1

TAG 46:2; 12:0-16:0-18:2

TAG 46:3; 12:0-16:1-18:2

TAG 47:0; 14:0-16:0-17:0

TAG 47:1; 14:0-15:0-18:1

TAG 47:2; 14:0-15:0-18:2

TAG 48:0; 14:0-16:0-18:0

TAG 48:1; 14:0-16:0-18:1

TAG 48:2; 14:0-16:0-18:2

TAG 48:3; 12:0-18:1-18:2

TAG 48:4 (1); 12:0-18:1-18:3

TAG 48:4 (2); 14:1-16:1-18:2

TAG 48:5; 12:0-18:2-18:3

OC-FA, odd-chain fatty acid; PUFA, polyunsaturated fatty acid; VLC-FA, very-long chain fatty acid; TAG, triacylglycerol.
