# Supplementary material for: Exercise training is associated with changes in adipocyte size and lipid metabolism in older women: cross-sectional and longitudinal study
Source: BMC Geriatr. 2026 Jun 23;26:1132. doi: 10.1186/s12877-026-07752-9 (PMC13543450; doi:10.1186/s12877-026-07752-9)
Supplement: Supplementary file 2 [file 12877_2026_7752_MOESM3_ESM.docx]

**Suppl. Tab. 2. Short-term ET: Enrichment analysis for down-regulated proteins, Reactome 2024.**

| **Term** | **Overlap** | **Adjusted P-value** | **Odds Ratio** | **Combined Score** |
| --- | --- | --- | --- | --- |
| Processing of Capped Intron-Containing Pre-mRNA | 40/285 | 1.005668653139577E-12 | 5.473819650452125 | 190.79904567971775 |
| Metabolism of RNA | 65/761 | 4.3701290455977325E-11 | 3.1973522167487687 | 97.17323573124759 |
| mRNA Splicing - Major Pathway | 30/204 | 4.3530819924882717E-10 | 5.702118820108018 | 157.87822195154664 |
| mRNA Splicing | 30/215 | 1.2993968572167804E-9 | 5.360003721449504 | 141.00217827501754 |
| Immune System | 111/2150 | 4.962683269977985E-6 | 1.8890142226581659 | 33.69134896291647 |
| Membrane Trafficking | 46/633 | 1.2699907963598427E-5 | 2.607806304744388 | 43.5854229332159 |
| Vesicle-mediated Transport | 50/752 | 4.506744126084653E-5 | 2.372518548989137 | 36.28227930417602 |
| Metabolism | 105/2181 | 2.460789423113876E-4 | 1.7305484475313577 | 23.296130322269548 |
| Antigen processing-Cross Presentation | 13/96 | 0.0010776346294928906 | 5.056574122577266 | 60.00661402055294 |
| Signaling by Rho GTPases | 42/672 | 0.0013882712449434344 | 2.1978910369068543 | 25.294204650480705 |
| Signaling by Rho GTPases, Miro GTPases and RHOBTB3 | 42/688 | 0.002211536291051168 | 2.141625903899623 | 23.445363023064647 |
| Antigen Presentation Folding, Assembly and Peptide Loading of Class I MHC | 7/31 | 0.003465244829702307 | 9.351200331125828 | 96.4093797666357 |
| RHO GTPase Effectors | 23/291 | 0.003465244829702307 | 2.790784597421058 | 28.758050587778346 |
| Innate Immune System | 60/1149 | 0.003465244829702307 | 1.8298810577312474 | 18.759726180287974 |
| Platelet Degranulation | 14/128 | 0.003465244829702307 | 3.9650004408004937 | 40.39625235972894 |
| Response to Elevated Platelet Cytosolic Ca2+ | 14/133 | 0.004835576389335626 | 3.7974184648733864 | 37.07250066283932 |
| Platelet Activation, Signaling and Aggregation | 21/262 | 0.004835576389335626 | 2.8279625852732257 | 27.515591257743804 |
| Hemostasis | 41/707 | 0.0054009295203464236 | 2.0221352931879246 | 19.335858781408238 |
| Adaptive Immune System | 47/854 | 0.005460048983015738 | 1.918835192069393 | 18.22345709245577 |
| Butyrate Response Factor 1 (BRF1) Binds and Destabilizes mRNA | 5/17 | 0.007855649979328317 | 13.323019801980198 | 120.35065931429463 |
| Tristetraprolin (TTP, ZFP36) Binds and Destabilizes mRNA | 5/17 | 0.007855649979328317 | 13.323019801980198 | 120.35065931429463 |
| Formation of Fibrin Clot (Clotting Cascade) | 7/39 | 0.00950690988696442 | 7.010502897350993 | 61.379439618303834 |
| ER-Phagosome Pathway | 10/80 | 0.00950690988696442 | 4.592108390777276 | 40.18797056421326 |
| Transport of Mature Transcript to Cytoplasm | 10/83 | 0.012402304973974305 | 4.4027078157408885 | 37.172529271194165 |
| Nef Mediated Downregulation of MHC Class I Complex Cell Surface Expression | 4/11 | 0.013094355701708579 | 18.246175570722524 | 152.09300595001156 |
| Metabolism of Carbohydrates | 21/290 | 0.013094355701708579 | 2.5298972969567135 | 21.020320774699915 |
| Signaling by Receptor Tyrosine Kinases | 32/536 | 0.013327865212613442 | 2.0708939879924335 | 17.09180967562203 |
| RAC2 GTPase Cycle | 10/88 | 0.016646036985974205 | 4.1194163573531295 | 32.79465340417782 |
| RHO GTPases Activate PAKs | 5/21 | 0.016646036985974205 | 9.990202145214521 | 79.36386124225133 |
| mRNA 3'-End Processing | 8/58 | 0.016646036985974205 | 5.131409618573798 | 40.66987329276977 |
| Signal Transduction | 109/2613 | 0.01739663175700186 | 1.4641633147919504 | 11.49189349954531 |
| RHOG GTPase Cycle | 9/74 | 0.017782106015587284 | 4.444569384104268 | 34.64592373604632 |
| Common Pathway of Fibrin Clot Formation | 5/22 | 0.018744809331097175 | 9.402057852844107 | 72.50507710293252 |
| MHC Class II Antigen Presentation | 12/126 | 0.019338293503451818 | 3.3872243212371496 | 25.84124858807689 |
| Regulation of mRNA Stability by Proteins That Bind AU-rich Elements | 9/76 | 0.019338293503451818 | 4.31144939753062 | 32.746187450777875 |
| Asparagine N-linked Glycosylation | 21/306 | 0.019338293503451818 | 2.3858697591436218 | 18.11694954790424 |
| Class I MHC Mediated Antigen Processing & Presentation | 24/373 | 0.01958529276226266 | 2.2305638402249306 | 16.83786028659561 |
| Metabolism of Proteins | 89/2067 | 0.01958529276226266 | 1.5007796489352223 | 11.295883978301918 |
| Intra-Golgi and Retrograde Golgi-to-ER Traffic | 16/204 | 0.019659845961648742 | 2.746433041301627 | 20.58974034234198 |
| RAC3 GTPase Cycle | 10/94 | 0.020506361276087574 | 3.8239838364630376 | 28.41001138174804 |
| Axon Guidance | 31/541 | 0.021714781149351867 | 1.9785294117647059 | 14.53719940411474 |
| Signaling by MET | 9/79 | 0.022009061776172997 | 4.126032273374466 | 30.153628473924616 |
| Nervous System Development | 32/567 | 0.022009061776172997 | 1.947695833938631 | 14.191667357579092 |
| Gene and Protein Expression by JAK-STAT Signaling After Interleukin-12 Stimulation | 6/37 | 0.025152328468598443 | 6.192908557717942 | 43.98862771258743 |
| Neutrophil Degranulation | 28/478 | 0.025152328468598443 | 2.0213150371640936 | 14.339444546008954 |
| Basigin Interactions | 5/25 | 0.025152328468598443 | 7.990511551155116 | 56.616373356757286 |
| Cytokine Signaling in Immune System | 40/776 | 0.026734869235957782 | 1.7753940455341506 | 12.403373683234815 |
| Toll-like Receptor Cascades | 14/174 | 0.026734869235957782 | 2.818320770519263 | 19.641170387311803 |
| ER to Golgi Anterograde Transport | 13/155 | 0.026734869235957782 | 2.946570728720147 | 20.45790661507744 |
| RNA Polymerase II Transcription Termination | 8/67 | 0.026734869235957782 | 4.346628439722292 | 30.170167942167122 |
| Interferon Signaling | 19/280 | 0.028345799102978884 | 2.3521279900590244 | 16.14203769411401 |
| Cellular Responses to Stimuli | 44/887 | 0.029478688131606128 | 1.7072310405643738 | 11.584190940252503 |
| Integrin Cell Surface Interactions | 9/85 | 0.029478688131606128 | 3.799112607099143 | 25.743268848650214 |
| Respiratory Electron Transport | 13/158 | 0.029478688131606128 | 2.8851574212893554 | 19.522102258910973 |
| Postmitotic Nuclear Pore Complex (NPC) Reformation | 5/27 | 0.030394188058171172 | 7.263351335133513 | 48.79125528310593 |
| Disease | 89/2131 | 0.03409517014567887 | 1.4483987601367452 | 9.537032121903524 |
| Synthesis of PG | 3/8 | 0.03434309336444027 | 19.128947368421052 | 125.47805346685179 |
| RHO GTPase Cycle | 26/450 | 0.036296377266839236 | 1.9879454926624738 | 12.895566513903 |
| Transport of Mature mRNA Derived From an Intronless Transcript | 6/42 | 0.0382179639744643 | 5.331404958677686 | 34.21801889787534 |
| Host Interactions of HIV Factors | 11/126 | 0.03878212481358856 | 3.0726666666666667 | 19.624317825656938 |
| Transport to the Golgi and Subsequent Modification | 14/186 | 0.04029306073268374 | 2.620057652604106 | 16.590176642461508 |
| Transport of Mature mRNAs Derived From Intronless Transcripts | 6/43 | 0.040707479778343325 | 5.187044896135805 | 32.64546882901264 |
| Transport of Mature mRNA Derived From an Intron-Containing Transcript | 8/74 | 0.040707479778343325 | 3.884215287200362 | 24.429755358440268 |
| MET Activates PTK2 Signaling | 5/30 | 0.042758321331680754 | 6.3907590759075905 | 39.77988937050274 |
| Nuclear Envelope (NE) Reassembly | 8/75 | 0.04300977273832125 | 3.8260439098042127 | 23.733821373550086 |
| Smooth Muscle Contraction | 6/44 | 0.043667991504830125 | 5.050282731622445 | 31.174244683286418 |
| Cellular Responses to Stress | 39/787 | 0.044599655187271936 | 1.6991674769081186 | 10.427151130654533 |
| Extracellular Matrix Organization | 19/300 | 0.046301357158831796 | 2.1824324324324325 | 13.248395203350965 |
| RHO GTPases Activate ROCKs | 4/19 | 0.046301357158831796 | 8.511367380560133 | 51.6356069097943 |
| GPER1 Signaling | 6/45 | 0.046301357158831796 | 4.920534011443102 | 29.79568242004769 |
