# Supplementary material for: Exercise training is associated with changes in adipocyte size and lipid metabolism in older women: cross-sectional and longitudinal study
Source: BMC Geriatr. 2026 Jun 23;26:1132. doi: 10.1186/s12877-026-07752-9 (PMC13543450; doi:10.1186/s12877-026-07752-9)
Supplement: Supplementary file 3 [file 12877_2026_7752_MOESM4_ESM.docx]

**Suppl. Tab. 3. Short-term ET: Enrichment analysis for up-regulated proteins, Reactome 2024.**

| **Term** | **Overlap** | **Adjusted P-value** | **Odds Ratio** | **Combined Score** |
| --- | --- | --- | --- | --- |
| Metabolism of Proteins | 152/2067 | 1.5258991995011684E-10 | 2.0904476851557026 | 62.459314425621976 |
| Metabolism of RNA | 74/761 | 1.5684227432541317E-9 | 2.715613832601565 | 72.92840822277797 |
| Metabolism | 153/2181 | 1.8161156858764575E-9 | 1.9769727629974254 | 52.00056345812572 |
| Processing of Capped Intron-Containing Pre-mRNA | 38/285 | 3.0432363602992125E-8 | 3.785214785214785 | 87.16585376235994 |
| Post-translational Protein Modification | 110/1457 | 3.0432363602992125E-8 | 2.0877871445193925 | 47.963764622874805 |
| mRNA Splicing - Major Pathway | 30/204 | 2.1486977924920541E-7 | 4.214608633986349 | 87.81831032841482 |
| mRNA Splicing | 30/215 | 6.582293951548625E-7 | 3.96171750156326 | 77.50297850650415 |
| Neutrophil Degranulation | 47/478 | 3.354978134772667E-6 | 2.6883877911284153 | 47.85544286067018 |
| Innate Immune System | 83/1149 | 2.8593823275069616E-5 | 1.9466364753833214 | 30.251251698678484 |
| Immune System | 129/2150 | 3.497122570497333E-4 | 1.6141697740732617 | 20.861203235224142 |
| Asparagine N-linked Glycosylation | 31/306 | 3.497122570497333E-4 | 2.7444811044811046 | 35.227272745404825 |
| Host Interactions of HIV Factors | 18/126 | 3.796676606255359E-4 | 4.026160337552743 | 50.99727913020417 |
| Disease | 126/2131 | 7.489611996457903E-4 | 1.5836961847580462 | 18.85714386143451 |
| RHOC GTPase Cycle | 13/74 | 7.653405960521904E-4 | 5.12842561088772 | 60.57339695458354 |
| Cytokine Signaling in Immune System | 57/776 | 9.707427662937264E-4 | 1.950039724502703 | 22.434370774194946 |
| TCR Signaling | 16/113 | 0.0010513466725285139 | 3.9768822243049047 | 44.94741800976414 |
| Signaling by Rho GTPases | 51/672 | 0.0010513466725285139 | 2.0147352567661567 | 22.765787865044377 |
| Extracellular Matrix Organization | 29/300 | 0.0011002253301695712 | 2.599173886475707 | 29.103056147002896 |
| Signaling by ROBO Receptors | 23/210 | 0.00110824786242929 | 2.9777240369222384 | 33.159066432097426 |
| RHO GTPase Cycle | 38/450 | 0.0011596581398096062 | 2.249527171857269 | 24.832688075081677 |
| Signaling by Rho GTPases, Miro GTPases and RHOBTB3 | 51/688 | 0.0015993554304792503 | 1.9624374493217671 | 20.936861428641592 |
| Antigen processing-Cross Presentation | 14/96 | 0.0019990459145771597 | 4.109172451926031 | 42.73216829706776 |
| Protein Localization | 19/164 | 0.002233030311070432 | 3.163262095188147 | 32.31772369201665 |
| Proteasome Assembly | 10/52 | 0.002233030311070432 | 5.713688984365676 | 58.28827942937161 |
| Nuclear Envelope Breakdown | 10/53 | 0.0024856774489707125 | 5.580521070117153 | 55.96650033894664 |
| CLEC7A (Dectin-1) Signaling | 13/87 | 0.0024856774489707125 | 4.224613292537821 | 42.30648500201058 |
| Membrane Trafficking | 47/633 | 0.00248915959475963 | 1.960959398671588 | 19.560855499923687 |
| Regulation of mRNA Stability by Proteins That Bind AU-rich Elements | 12/76 | 0.0025840576129828687 | 4.505653266331659 | 44.612104967038356 |
| ER to Golgi Anterograde Transport | 18/155 | 0.00280524048272094 | 3.169084357387046 | 31.00677294579612 |
| Nervous System Development | 43/567 | 0.002894691276219032 | 2.002504615077583 | 19.462045486180543 |
| Vesicle-mediated Transport | 53/752 | 0.0028952003324217777 | 1.8572018683265592 | 17.988645735065084 |
| Transport to the Golgi and Subsequent Modification | 20/186 | 0.0030305193860031675 | 2.9089963916580026 | 27.95096870970064 |
| Downstream TCR Signaling | 13/91 | 0.0031635827468913125 | 4.007127882599581 | 38.20682181202741 |
| NIK-->noncanonical NF-kB Signaling | 9/46 | 0.0033429576918040677 | 5.831444711294523 | 55.10546794691892 |
| ER-Phagosome Pathway | 12/80 | 0.00346382066254902 | 4.23972805202483 | 39.790726745988906 |
| COPII-mediated Vesicle Transport | 11/69 | 0.0037563208904083465 | 4.5531519058538485 | 42.23489442026013 |
| Class I Peroxisomal Membrane Protein Import | 6/20 | 0.0039195032770719795 | 10.248307801923762 | 94.2208310482286 |
| Translation | 27/302 | 0.0039195032770719795 | 2.37811197765103 | 21.829585220966802 |
| Regulation of Ornithine Decarboxylase (ODC) | 8/38 | 0.004222797368090626 | 6.387333333333333 | 57.989757230077004 |
| Dectin-1 Mediated Noncanonical NF-kB Signaling | 9/49 | 0.004754899844121362 | 5.393241551939925 | 48.18792569204273 |
| HIV Infection | 22/227 | 0.004937548048269662 | 2.592403649227332 | 23.001066821691445 |
| Signaling by Receptor Tyrosine Kinases | 40/536 | 0.005208655193431791 | 1.9632056451612903 | 17.26626854973991 |
| Cell Cycle | 46/649 | 0.005555900686580298 | 1.8609794422463362 | 16.203300368677276 |
| Axon Guidance | 40/541 | 0.006034271621489906 | 1.9430929807052562 | 16.713092252453873 |
| ISG15 Antiviral Mechanism | 11/76 | 0.0071795075334839965 | 4.0613261268217355 | 34.06641252365438 |
| Infectious Disease | 79/1311 | 0.0071795075334839965 | 1.5797748205155613 | 13.225479171450912 |
| AUF1 (hnRNP D0) Binds and Destabilizes mRNA | 8/42 | 0.0071795075334839965 | 5.634705882352941 | 46.9962407204773 |
| Vif-mediated Degradation of APOBEC3G | 8/42 | 0.0071795075334839965 | 5.634705882352941 | 46.9962407204773 |
| Mitotic Anaphase | 21/221 | 0.007616428864291868 | 2.5338754764930114 | 20.93183775947403 |
| Mitotic Metaphase and Anaphase | 21/222 | 0.007936435408820993 | 2.5211363765669743 | 20.67131234622108 |
| Hh Mutants Are Degraded by ERAD | 8/43 | 0.007936435408820993 | 5.473428571428571 | 44.71942331111941 |
| Cell Cycle, Mitotic | 38/516 | 0.007936435408820993 | 1.9321088952888117 | 15.766432966229408 |
| Disorders of Transmembrane Transporters | 17/164 | 0.009710632578072656 | 2.784428562828418 | 22.106731831069833 |
| Complex I Biogenesis | 10/68 | 0.010099720101597398 | 4.134042001555613 | 32.54472502287071 |
| Diseases of Signal Transduction by Growth Factor Receptors and Second Messengers | 34/451 | 0.010099720101597398 | 1.9777976068757397 | 15.546894058269286 |
| Regulation of RUNX3 Expression and Activity | 8/45 | 0.010099720101597398 | 5.177027027027027 | 40.614130084478674 |
| Interferon Signaling | 24/280 | 0.011012109984303698 | 2.264349489795918 | 17.4254333593932 |
| Protein Lipoylation | 4/10 | 0.011012109984303698 | 15.908789386401327 | 122.35856094727247 |
| Hh Mutants Abrogate Ligand Secretion | 8/46 | 0.011012109984303698 | 5.0405263157894735 | 38.759567956272164 |
| Metabolism of Polyamines | 8/46 | 0.011012109984303698 | 5.0405263157894735 | 38.759567956272164 |
| Degradation of Beta-Catenin by the Destruction Complex | 10/72 | 0.013483986781118283 | 3.8665211415635863 | 28.632621453408365 |
| Syndecan Interactions | 6/27 | 0.013483986781118283 | 6.829711435696473 | 50.55725311086515 |
| Cross-presentation of Soluble Exogenous Antigens (Endosomes) | 7/37 | 0.013483986781118283 | 5.5819392426134 | 41.31682624059659 |
| Regulation of Activated PAK-2p34 by Proteasome Mediated Degradation | 7/37 | 0.013483986781118283 | 5.5819392426134 | 41.31682624059659 |
| Integrin Cell Surface Interactions | 11/85 | 0.013483986781118283 | 3.5657024653260536 | 26.39166453306851 |
| Defective CFTR Causes Cystic Fibrosis | 8/48 | 0.013483986781118283 | 4.788 | 35.39179494852968 |
| Nuclear Events Mediated by NFE2L2 | 11/87 | 0.015858000692690256 | 3.471504985802021 | 25.00315081433982 |
| FGFR2 Alternative Splicing | 6/28 | 0.015858000692690256 | 6.518929947857629 | 46.90896923973192 |
| Glycolysis | 10/74 | 0.015858000692690256 | 3.7453007518796992 | 26.910536256623065 |
| Metabolism of Lipids | 49/758 | 0.01614616466102178 | 1.682986113046823 | 11.922706105812752 |
| C-type Lectin Receptors (CLRs) | 14/131 | 0.01614616466102178 | 2.8746582273030636 | 20.32669999459188 |
| p53-Independent DNA Damage Response | 7/39 | 0.01614616466102178 | 5.232521847690387 | 36.99660704387698 |
| p53-Independent G1 S DNA Damage Checkpoint | 7/39 | 0.01614616466102178 | 5.232521847690387 | 36.99660704387698 |
| Autodegradation of the E3 Ubiquitin Ligase COP1 | 7/39 | 0.01614616466102178 | 5.232521847690387 | 36.99660704387698 |
| Ubiquitin Mediated Degradation of Phosphorylated Cdc25A | 7/39 | 0.01614616466102178 | 5.232521847690387 | 36.99660704387698 |
| Ubiquitin-dependent Degradation of Cyclin D | 7/39 | 0.01614616466102178 | 5.232521847690387 | 36.99660704387698 |
| ABC-family Proteins Mediated Transport | 11/90 | 0.017661535296140358 | 3.3391515652049617 | 23.092968956617206 |
| Regulation of Apoptosis | 7/40 | 0.017661535296140358 | 5.073695759089017 | 35.07513835142607 |
| Vpu Mediated Degradation of CD4 | 7/40 | 0.017661535296140358 | 5.073695759089017 | 35.07513835142607 |
| PECAM1 Interactions | 4/12 | 0.017661535296140358 | 11.930348258706468 | 82.3026720127085 |
| Regulation of Expression of SLITs and ROBOs | 16/164 | 0.017661535296140358 | 2.5995085995085994 | 17.91513497146788 |
| KEAP1-NFE2L2 Pathway | 13/119 | 0.017661535296140358 | 2.944321822712709 | 20.203087428421405 |
| RHOA GTPase Cycle | 15/149 | 0.017661535296140358 | 2.6902373379006606 | 18.455903551610508 |
| Beta-catenin Independent WNT Signaling | 14/134 | 0.017661535296140358 | 2.802350965575147 | 19.191021458052365 |
| Hedgehog Ligand Biogenesis | 8/52 | 0.017661535296140358 | 4.351818181818182 | 29.781361295533827 |
| Mitochondrial Translation Elongation | 11/91 | 0.017661535296140358 | 3.2972396486825595 | 22.49830132678927 |
| Mitochondrial Translation Initiation | 11/91 | 0.017661535296140358 | 3.2972396486825595 | 22.49830132678927 |
| Mitochondrial Translation Termination | 11/91 | 0.017661535296140358 | 3.2972396486825595 | 22.49830132678927 |
| Cellular Responses to Stimuli | 55/887 | 0.017661535296140358 | 1.6118219429972418 | 10.997303321224187 |
| SARS-CoV Infections | 35/498 | 0.017713266300977387 | 1.8315642122498246 | 12.447250027996224 |
| Costimulation by the CD28 Family | 10/78 | 0.017713266300977387 | 3.524251805985552 | 23.85652737129837 |
| ABC Transporter Disorders | 9/65 | 0.017713266300977387 | 3.8490970856427675 | 26.040369449256684 |
| Signaling by BRAF and RAF1 Fusions | 9/65 | 0.017713266300977387 | 3.8490970856427675 | 26.040369449256684 |
| The Role of GTSE1 in G2 M Progression After G2 Checkpoint | 9/65 | 0.017713266300977387 | 3.8490970856427675 | 26.040369449256684 |
| Methionine Salvage Pathway | 3/6 | 0.018222117938200447 | 23.83726708074534 | 160.33934082179246 |
| Oxygen-dependent Proline Hydroxylation of Hypoxia-inducible Factor Alpha | 8/53 | 0.018235034631445247 | 4.254888888888889 | 28.57257744124938 |
| M Phase | 28/372 | 0.01856559902017861 | 1.966845557543232 | 13.152111884408773 |
| PCP CE Pathway | 10/79 | 0.018634597264955505 | 3.4729940794014023 | 23.16492079368245 |
| Signaling by CSF1 (M-CSF) in Myeloid Cells | 6/31 | 0.018634597264955505 | 5.735760598503741 | 38.02150154924776 |
| Degradation of AXIN | 7/42 | 0.018634597264955505 | 4.783270911360799 | 31.634242427679666 |
| FBXL7 Down-Regulates AURKA During Mitotic Entry and in Early Mitosis | 7/42 | 0.018634597264955505 | 4.783270911360799 | 31.634242427679666 |
| GSK3B and BTRC CUL1-mediated-degradation of NFE2L2 | 7/42 | 0.018634597264955505 | 4.783270911360799 | 31.634242427679666 |
| SCF-beta-TrCP Mediated Degradation of Emi1 | 7/42 | 0.018634597264955505 | 4.783270911360799 | 31.634242427679666 |
| Transport of Mature mRNA Derived From an Intronless Transcript | 7/42 | 0.018634597264955505 | 4.783270911360799 | 31.634242427679666 |
| Activation of NF-kappaB in B Cells | 8/54 | 0.018869018201189146 | 4.162173913043478 | 27.42999976899519 |
| Signaling by VEGF | 12/108 | 0.018869018201189146 | 2.998743718592965 | 19.727576518390258 |
| MAPK6 MAPK4 Signaling | 10/80 | 0.018869018201189146 | 3.423200859291085 | 22.499252725165668 |
| Metabolism of Amino Acids and Derivatives | 27/358 | 0.01962034700986475 | 1.9699239103945287 | 12.852235158632908 |
| Transport of Mature mRNAs Derived From Intronless Transcripts | 7/43 | 0.020367357608728695 | 4.650159522818699 | 30.090052697018084 |
| Regulation of RAS by GAPs | 8/55 | 0.020367357608728695 | 4.073404255319149 | 26.348871355679254 |
| Insertion of Tail-Anchored Proteins Into the Endoplasmic Reticulum Membrane | 5/22 | 0.02052036628844855 | 7.023294996703538 | 45.31415552846041 |
| Downstream Signaling Events of B Cell Receptor (BCR) | 9/68 | 0.020585456352732893 | 3.6528075348422817 | 23.523510331998057 |
| Intra-Golgi and Retrograde Golgi-to-ER Traffic | 18/204 | 0.020726109964709305 | 2.3282155982033483 | 14.95679581255422 |
| Mitotic Prophase | 12/111 | 0.021572552649264123 | 2.907415867214862 | 18.43790493373336 |
| MHC Class II Antigen Presentation | 13/126 | 0.021572552649264123 | 2.76091723715701 | 17.495368951732992 |
| Degradation of DVL | 7/44 | 0.021572552649264123 | 4.524243344468063 | 28.649238517717112 |
| Elastic Fibre Formation | 7/44 | 0.021572552649264123 | 4.524243344468063 | 28.649238517717112 |
| Negative Regulation of NOTCH4 Signaling | 7/44 | 0.021572552649264123 | 4.524243344468063 | 28.649238517717112 |
| Stabilization of P53 | 7/44 | 0.021572552649264123 | 4.524243344468063 | 28.649238517717112 |
| Cellular Responses to Stress | 49/787 | 0.021940705463167447 | 1.6143156556730258 | 10.16831339565378 |
| Mitochondrial Translation | 11/97 | 0.021940705463167447 | 3.0662367599428086 | 19.313065057977855 |
| Transport of Mature Transcript to Cytoplasm | 10/83 | 0.021940705463167447 | 3.2820063858275828 | 20.64566775650033 |
| Antiviral Mechanism by IFN-stimulated Genes | 15/158 | 0.022010091249708437 | 2.519731214560975 | 15.801599786046047 |
| Respiratory Electron Transport | 15/158 | 0.022010091249708437 | 2.519731214560975 | 15.801599786046047 |
| Somitogenesis | 7/45 | 0.023321700359994025 | 4.404954333399041 | 27.3026919432248 |
| APEX1-Independent Resolution of AP Sites via the Single Nucleotide Replacement Pathway | 3/7 | 0.023321700359994025 | 17.877018633540374 | 110.78855785406529 |
| Aerobic Respiration and Respiratory Electron Transport | 21/260 | 0.023820150313566893 | 2.116043659253667 | 13.047927865955623 |
| SARS-CoV-2 Infection | 24/313 | 0.023820150313566893 | 2.0022950356613234 | 12.334869178847601 |
| VEGFA-VEGFR2 Pathway | 11/99 | 0.024258498944982436 | 2.9962358845671266 | 18.37995410379521 |
| Orc1 Removal From Chromatin | 8/58 | 0.024401061624714525 | 3.8284 | 23.43274249699546 |
| Glucose Metabolism | 10/85 | 0.024459909384621708 | 3.1941520467836257 | 19.51848846117381 |
| Separation of Sister Chromatids | 16/177 | 0.024608302177369302 | 2.387979170587866 | 14.559608827674783 |
| Transcriptional Regulation by RUNX3 | 10/86 | 0.02629814205416529 | 3.15195884447962 | 18.984500837594215 |
| tRNA Processing in the Nucleus | 8/59 | 0.0264359259206974 | 3.7531372549019606 | 22.55772060515245 |
| Depolymerization of the Nuclear Lamina | 4/15 | 0.02678738648757822 | 8.675260063319765 | 51.8874560934495 |
| Transport of the SLBP Independent Mature mRNA | 6/35 | 0.02678738648757822 | 4.943589302605555 | 29.539853399292834 |
| Cellular Response to Heat Stress | 11/101 | 0.02678738648757822 | 2.9293461592081416 | 17.502888850640648 |
| Signal Transduction | 133/2613 | 0.026914570152031837 | 1.3277753882915173 | 7.894266573596051 |
| Degradation of GLI1 by the Proteasome | 7/47 | 0.026914570152031837 | 4.1842696629213485 | 24.860837895725158 |
| Degradation of GLI2 by the Proteasome | 7/47 | 0.026914570152031837 | 4.1842696629213485 | 24.860837895725158 |
| GLI3 Is Processed to GLI3R by the Proteasome | 7/47 | 0.026914570152031837 | 4.1842696629213485 | 24.860837895725158 |
| Viral Infection Pathways | 60/1029 | 0.028166658204266115 | 1.5085015148755816 | 8.88350748797494 |
| TNFR2 Non-Canonical NF-kB Pathway | 10/88 | 0.028984960555798432 | 3.070818070818071 | 17.971346656870214 |
| Transport of Mature mRNA Derived From an Intron-Containing Transcript | 9/74 | 0.028984960555798432 | 3.3145855396168287 | 19.378184759141448 |
| Transport of the SLBP Dependant Mature mRNA | 6/36 | 0.029339693998343682 | 4.778553615960099 | 27.845838566431084 |
| Neddylation | 19/234 | 0.030998517393880248 | 2.1255224452500956 | 12.243155320849905 |
| Nuclear Envelope (NE) Reassembly | 9/75 | 0.030998517393880248 | 3.2641938787120264 | 18.782629734168697 |
| Signaling by WNT | 22/287 | 0.030998517393880248 | 1.999107014258966 | 11.49841564075983 |
| G2 M Transition | 16/184 | 0.03214863658662472 | 2.2876382876382877 | 13.059237666083439 |
| Cellular Response to Hypoxia | 8/62 | 0.032393663947727434 | 3.544074074074074 | 20.181125400742467 |
| Molecules Associated With Elastic Fibres | 6/37 | 0.03249017707033865 | 4.62416539296919 | 26.285973865292704 |
| Apoptosis | 15/168 | 0.03249017707033865 | 2.35380659199367 | 13.36517104844603 |
| Adaptive Immune System | 51/854 | 0.033059932816971206 | 1.5428256982155752 | 8.718767836342568 |
| Transcriptional Regulation by RUNX1 | 17/202 | 0.033059932816971206 | 2.2080773567499232 | 12.47045456628908 |
| Metabolism of Carbohydrates | 22/290 | 0.0335328174990393 | 1.97641563176484 | 11.121247072002264 |
| Mitotic G2-G2 M Phases | 16/186 | 0.03415122962108162 | 2.2604872251931076 | 12.663866547059015 |
| rRNA Modification in the Nucleus and Cytosol | 8/63 | 0.03422413402059097 | 3.4794545454545456 | 19.463201385446013 |
| SCF(Skp2)-mediated Degradation of P27 P21 | 7/50 | 0.03436781049007864 | 3.8917341694974303 | 21.728377619089176 |
| SARS-CoV-2 Activates Modulates Innate and Adaptive Immune Responses | 13/138 | 0.0357615655555047 | 2.4942993710691823 | 13.809333705611333 |
| Post-translational Protein Phosphorylation | 11/107 | 0.0357615655555047 | 2.7453994144709326 | 15.16736951505049 |
| Sphingolipid Metabolism | 11/107 | 0.0357615655555047 | 2.7453994144709326 | 15.16736951505049 |
| Mitotic G1 Phase and G1 S Transition | 13/139 | 0.03729580662230386 | 2.4743735649396026 | 13.54652692276196 |
| Asymmetric Localization of PCP Proteins | 7/51 | 0.03729580662230386 | 3.8030870502780614 | 20.804013781445136 |
| Regulation of IGF Transport and Uptake by Insulin-like Growth Factor Binding Proteins (IGFBPs) | 12/124 | 0.03898525447277641 | 2.5681981335247666 | 13.91873146797929 |
| Glycosphingolipid Catabolism | 6/39 | 0.03898525447277641 | 4.343459532985718 | 23.51461531405217 |
| Signaling by TGF-beta Receptor Complex | 10/94 | 0.04036148128868363 | 2.850578828022437 | 15.316342233053824 |
| Autophagy | 14/158 | 0.04250492779753749 | 2.3323537643436887 | 12.39719207693645 |
| Platelet Activation, Signaling and Aggregation | 20/262 | 0.04294229864332329 | 1.9874564752275874 | 10.531743121892568 |
| Vpr-mediated Nuclear Import of PICs | 6/40 | 0.043279714095207984 | 4.2154906850520755 | 22.28032200323985 |
| MECP2 Regulates Neuronal Receptors and Channels | 4/18 | 0.043431641526418256 | 6.815209665955934 | 35.917726880331486 |
| Hemostasis | 43/707 | 0.043431641526418256 | 1.568438459721238 | 8.265789568856471 |
| p53-Dependent G1 DNA Damage Response | 7/53 | 0.04365023080771301 | 3.637355479563589 | 19.108583063456756 |
| p53-Dependent G1 S DNA Damage Checkpoint | 7/53 | 0.04365023080771301 | 3.637355479563589 | 19.108583063456756 |
| Diseases of Metabolism | 20/264 | 0.045056166047977644 | 1.9709578097694933 | 10.280441932840963 |
| Signaling by Interleukins | 30/452 | 0.04594327656225288 | 1.7151159249016192 | 8.902710729103044 |
| Phosphorylation of CD3 and TCR Zeta Chains | 5/29 | 0.04621577155998937 | 4.973017849730178 | 25.755868577803994 |
| Generation of Second Messenger Molecules | 6/41 | 0.046302574772692374 | 4.094834342714642 | 21.134073408363026 |
| Oncogenic MAPK Signaling | 9/82 | 0.046302574772692374 | 2.950108868962916 | 15.21010994222951 |
| snRNP Assembly | 7/54 | 0.046302574772692374 | 3.559778999654687 | 18.330131486984797 |
| Autodegradation of Cdh1 by Cdh1 APC C | 7/54 | 0.046302574772692374 | 3.559778999654687 | 18.330131486984797 |
| Metabolism of Non-Coding RNA | 7/54 | 0.046302574772692374 | 3.559778999654687 | 18.330131486984797 |
| Initiation of Nuclear Envelope (NE) Reformation | 4/19 | 0.0499200237887121 | 6.360530679933665 | 32.22168704775294 |
| Diseases of Glycosylation | 13/146 | 0.0499200237887121 | 2.343282735139736 | 11.864085584478712 |
| APC C Cdc20 Mediated Degradation of Securin | 7/55 | 0.049958716374401586 | 3.4854348730753224 | 17.59348667279766 |
| G1 S DNA Damage Checkpoints | 7/55 | 0.049958716374401586 | 3.4854348730753224 | 17.59348667279766 |
| NS1 Mediated Effects on Host Pathways | 6/42 | 0.049958716374401586 | 3.9808811305070657 | 20.067646782157013 |
| Laminin Interactions | 5/30 | 0.049958716374401586 | 4.773848069738481 | 24.01237745916717 |
| PD-1 Signaling | 5/30 | 0.049958716374401586 | 4.773848069738481 | 24.01237745916717 |
| Regulation of TP53 Activity Through Acetylation | 5/30 | 0.049958716374401586 | 4.773848069738481 | 24.01237745916717 |
